# Structural modeling of hERG channel–drug interactions using Rosetta

**DOI:** 10.3389/fphar.2023.1244166

**Published:** 2023-11-14

**Authors:** Aiyana M. Emigh Cortez, Kevin R. DeMarco, Kazuharu Furutani, Slava Bekker, Jon T. Sack, Heike Wulff, Colleen E. Clancy, Igor Vorobyov, Vladimir Yarov-Yarovoy

**Affiliations:** ^1^ Biophysics Graduate Group, University of California, Davis, Davis, CA, United States; ^2^ Department of Physiology and Membrane Biology, University of California, Davis, Davis, CA, United States; ^3^ Department of Pharmacology, Tokushima Bunri University, Tokushima, Japan; ^4^ American River College, Sacramento, CA, United States; ^5^ Department of Anesthesiology and Pain Medicine, University of California, Davis, Davis, CA, United States; ^6^ Department of Pharmacology, University of California, Davis, Davis, CA, United States; ^7^ Center for Precision Medicine and Data Sciences, University of California, Davis, Davis, CA, United States

**Keywords:** hERG channel, potassium channel, state-dependent, drug block, Rosetta

## Abstract

The human ether-a-go-go-related gene (hERG) not only encodes a potassium-selective voltage-gated ion channel essential for normal electrical activity in the heart but is also a major drug anti-target. Genetic hERG mutations and blockage of the channel pore by drugs can cause long QT syndrome, which predisposes individuals to potentially deadly arrhythmias. However, not all hERG-blocking drugs are proarrhythmic, and their differential affinities to discrete channel conformational states have been suggested to contribute to arrhythmogenicity. We used Rosetta electron density refinement and homology modeling to build structural models of open-state hERG channel wild-type and mutant variants (Y652A, F656A, and Y652A/F656 A) and a closed-state wild-type channel based on cryo-electron microscopy structures of hERG and EAG1 channels. These models were used as protein targets for molecular docking of charged and neutral forms of amiodarone, nifekalant, dofetilide, d/l-sotalol, flecainide, and moxifloxacin. We selected these drugs based on their different arrhythmogenic potentials and abilities to facilitate hERG current. Our docking studies and clustering provided atomistic structural insights into state-dependent drug–channel interactions that play a key role in differentiating safe and harmful hERG blockers and can explain hERG channel facilitation through drug interactions with its open-state hydrophobic pockets.

## 1 Introduction

The human ether-à-go-go-related gene (hERG) encodes voltage-gated potassium channel Kv11.1 that mediates the rapid repolarization phase during cardiac action potential (Sanguinetti and Tristani-Firouzi, 2006; Vandenberg et al., 2012). The hERG channel cycles between closed, open, and inactivated states in response to membrane voltage changes to tightly regulate K^+^ currents in the heart (Sanguinetti and Tristani-Firouzi, 2006; Vandenberg et al., 2012). Genetic mutations of hERG or drugs can result in long QT syndrome (LQTS), potentially leading to fatal arrhythmias such as torsade de pointes (TdP). The promiscuous block of the cardiac hERG channel by structurally varied drugs is a major research question and drug-design challenge.

A significant impediment to the development and approval of new drugs is that the drug safety guidelines developed by the International Council for Harmonization of Technical Requirements for Pharmaceuticals for Human Use (ICH) are not sufficiently selective (Huang et al., 2017). Although early testing of QT prolongation and hERG channel block are extremely effective at eliminating the risk of approving potentially torsadogenic drugs, they are inadequate markers of true proarrhythmic risk (Colatsky et al., 2016; Huang et al., 2017) since a number of hERG-blocking and QT-prolonging drugs demonstrate low proarrhythmic proclivities. Surrogate markers, such as hERG channel block in cell cultures or QT prolongation in animal models, often do not correlate with arrhythmogenicity in human subjects; however, multi-scale *in silico* models of drug cardiotoxicity assessment may provide better accuracy (Passini et al., 2017; Yang et al., 2020). The Comprehensive *in vitro* Proarrhythmia Assay (CiPA) initiative seeks to establish a new paradigm for the early safety assessment of drugs that differentiate effects on QT interval prolongation from TdP arrhythmia generation, removing the current constraints on drug development (Colatsky et al., 2016). Additionally, certain drugs, such as nifekalant and amiodarone, not only block hERG but also exhibit secondary effects on hERG current, such as “facilitation” that increases channel current potentials close to the threshold for channel activation (Hosaka et al., 2007; Furutani et al., 2011; Yamakawa et al., 2012) and has been postulated to lower risk for arrhythmia, complicating the cardiotoxicity assessment of the drugs. The goal of this study is to elucidate atomic-level interactions between open and closed states of the hERG channel and various drugs as a contribution to the multi-scale *in silico* models and experimental testing of proarrhythmic risk.

The hERG channel is a homotetramer, with each subunit composed of six transmembrane segments (S1–S6) (Vandenberg et al., 2012; Wang and MacKinnon, 2017). The S1–S4 segments of each subunit form the voltage-sensing domains; the S5 and S6 segments, along with intervening pore and turret helices and connecting loops, form the ion-conducting pore (Vandenberg et al., 2012; Wang and MacKinnon, 2017). Multiple laboratories have shown that Y652 and F656 on the S6 segment form canonical drug-interacting residues (Mitcheson et al., 2000a; Chen et al., 2002; Perry et al., 2004; Kamiya et al., 2006; Milnes et al., 2006; Du et al., 2014; Melgari et al., 2015; Saxena et al., 2016; Zhang et al., 2016; Helliwell et al., 2018; Kudaibergenova et al., 2020), as reviewed by Butler et al. (2019). Additionally, the interactions between drugs and the hERG channel are often shown to be protein conformational state-dependent (Vandenberg et al., 2017), typically showing preferential block for the open or inactivated states (Mitcheson et al., 2000a; Lees-Miller et al., 2000; Ficker et al., 2001; Weerapura et al., 2002; McPate et al., 2008; Perrin et al., 2008; Wu et al., 2015; Thouta et al., 2018). The MacKinnon laboratory solved the structure of a putatively open state of the hERG channel using single-particle cryo-electron microscopy (cryo-EM) at a resolution of 3.8 Å (Wang and MacKinnon, 2017). The homologous EAG1 channel structure in a closed state was also published by the MacKinnon laboratory (Whicher and MacKinnon, 2016) and can be used to build a closed-state model of the hERG channel. Potentially inactivated state structures of channels homologous to the hERG channel have not yet been resolved. Recently published cryo-EM hERG channel structures are inconclusive as to which state they represent (Asai et al., 2021).

**FIGURE 1:**
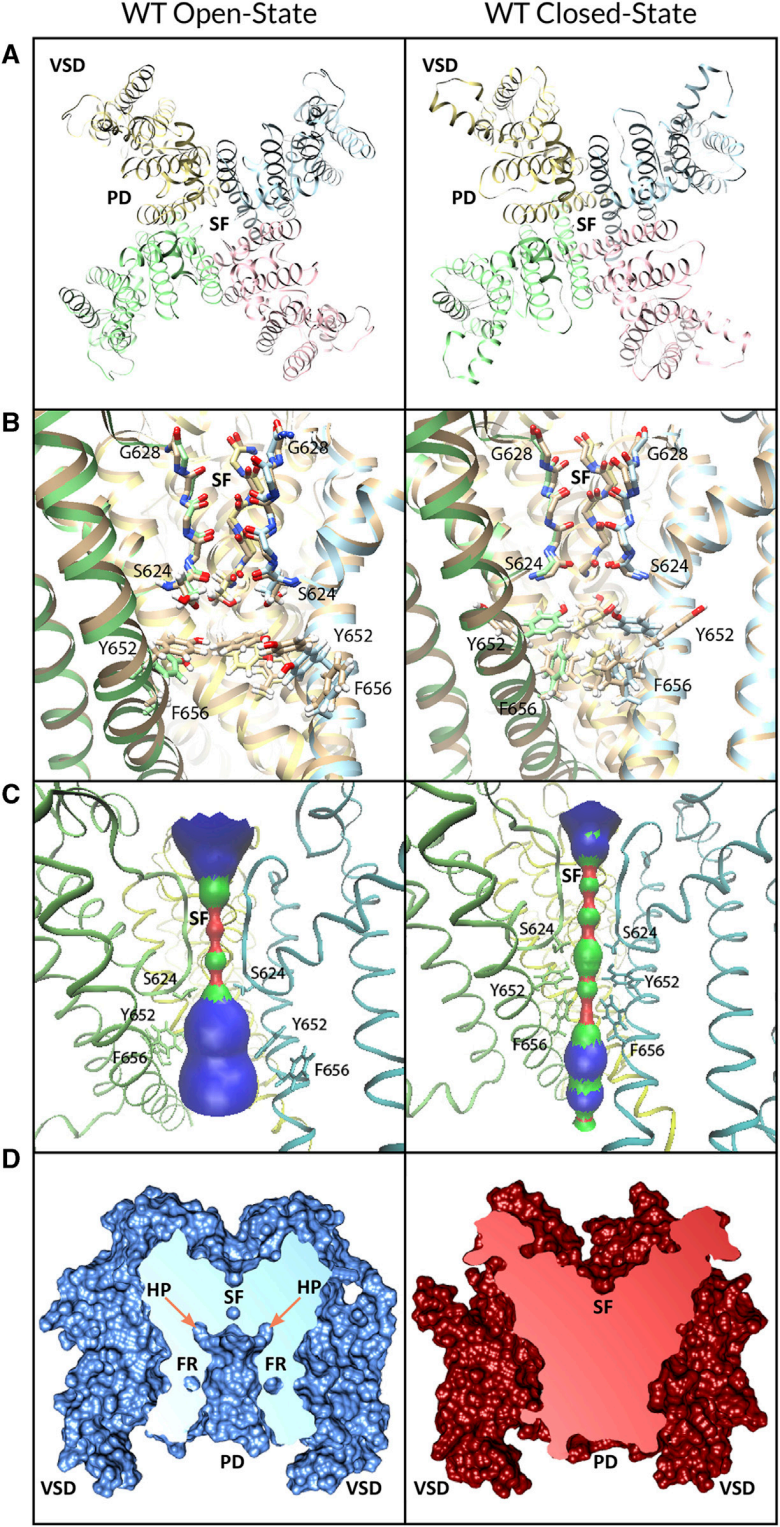
WT hERG channel models in open (*left panels*) and closed (*right panels*) states. **(A)** Extracellular view of the channel. Chain A is in pink, chain B is in blue, chain C is in yellow, and chain D is in green. Voltage-sensing domain (VSD), pore-forming domain (PD), and selectivity filter (SF) regions are labeled. **(B)** Comparison of the PD of cryo-EM-refined models (multi-colored) to the hERG structure (gold, PDB: 5VA2) (left panel) and closed-state hERG homology model (gold, based on PDB: 5K7L) (right panel). **(C)** HOLE profile of PD volume. **(D)** Depiction of the solvent-excluded molecular surface cross-section computed using UCSF Chimera. VSD, PD, SF, hydrophobic pocket (HP), and fenestration (FR) regions are labeled.

We previously studied molecular mechanisms of drug interactions with structural models of the hERG channel in inactivated states (Maly et al., 2022). In this study, we used Rosetta computational modeling software to study atomic-level interactions between the hERG channel in an open and closed state and drugs with relatively low (amiodarone and nifekalant), intermediate (flecainide and moxifloxacin), or high (d/l-sotalol and dofetilide) risk for arrhythmia (Doggrell, 2001; Kang et al., 2001; Pantazopoulos et al., 2011; Haverkamp et al., 2012; Barman, 2015; Furutani et al., 2019; Orvos et al., 2019; Mujovic et al., 2020; DeMarco et al., 2021). The structural models of an open-state wild-type (WT) hERG channel, its mutants (Y652A, F656A, and Y652A/F656A double mutant), and the closed-state WT hERG channel (Figure 1) were developed as targets for docking studies of cationic and neutral forms of the drugs (Table 1). We selected these drugs based on their different arrhythmogenic potentials (Colatsky et al., 2016; Crumb et al., 2016; Huang et al., 2017) and abilities to facilitate hERG current (Furutani et al., 2011; Furutani et al., 2019).

**TABLE 1:** Chemical and 3D structures of drugs in different ionization states selected for this study. 3D structures are shown in the stick representation, with C atoms in gray, O atoms in red, N atoms in blue, S atoms in yellow, and I atoms in violet. H atoms are not shown for clarity. The percentage of each drug formed in the aqueous phase was calculated for physiological pH 7.4 based on its p*K*_a_ and was rounded to the nearest whole number. Moxifloxacin percentages do not add up to 100% due to a cationic state with 7% prevalence not tested in this work.

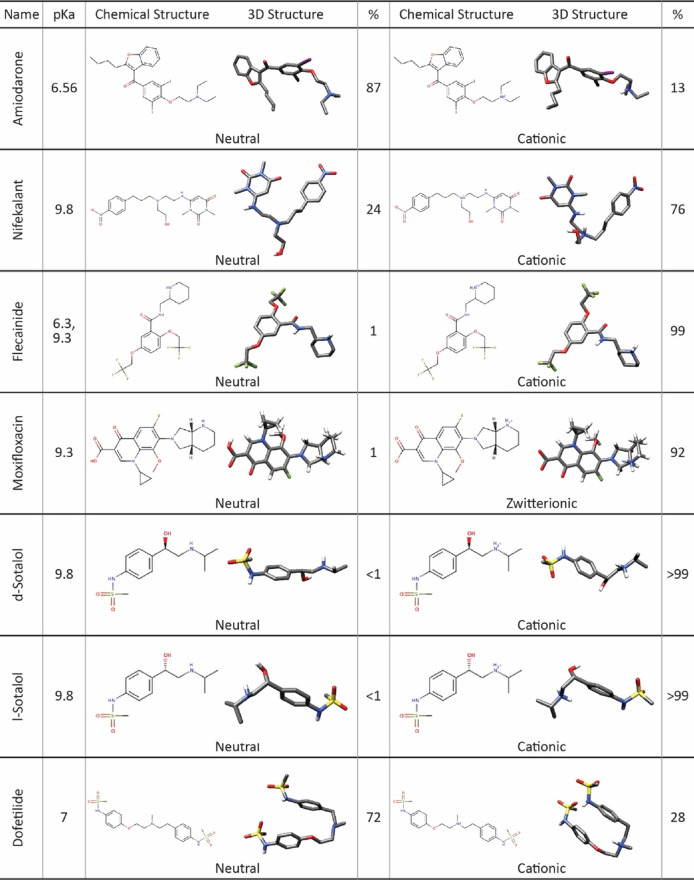

Amiodarone is an iodine-substituted, benzofuran-based class III antiarrhythmic drug targeting several K^+^ channels; it is not only used for the treatment of supraventricular and ventricular arrhythmias but also inhibits Na^+^ channels, beta-adrenoceptors, and Ca^2+^ channels (Kodama et al., 1997; Singh, 1997; Doggrell, 2001; Zimetbaum, 2012; Mujovic et al., 2020). It has high-affinity IC_50_ values of ∼45–220 nM and low-affinity IC_50_ values of ∼10–40 µM (Kamiya et al., 2001; Redfern et al., 2003; Lin et al., 2005; Waldhauser et al., 2008; Zhang et al., 2016). Experimental data suggest that F656 and Y652 in the S6 segment and S624 at the base of the selectivity filter (SF) play key roles in the ability of amiodarone to block hERG channels (Zhang et al., 2016). Nifekalant is a pyrimidinedione-based class III antiarrhythmic drug used in the treatment of ventricular tachycardia (Pantazopoulos et al., 2011). It is a high-affinity hERG blocker with reported IC_50_ values of ∼70–145 nM (Kushida et al., 2002; Ridley et al., 2004; Furutani et al., 2019). Experimental data suggest that G648, Y652, and F656 residues in the S6 segment and T623 and V625 at the base of the SF play key roles in the ability of nifekalant to block hERG channels (Hosaka et al., 2007). F656 and Y652 were also shown to be important for hERG current facilitation by nifekalant and amiodarone based on experimental mutagenesis studies corroborated by a 3D pharmacophore model (Hosaka et al., 2007). Flecainide is a class IC antiarrhythmic drug derived from trifluoroethoxy benzamide used in the treatment of ventricular tachycardia. Its therapeutic mechanism of action is inhibition of the cardiac voltage-gated sodium channel Na_V_1.5 (Ramos and O'Leary M, 2004). Flecainide inhibits hERG with reported IC_50_ values of ∼3 µM by interacting with F656 in the S6 segment (Melgari et al., 2015). Notably, V625 at the base of the SF attenuates hERG drug block, which was attributed to allosteric effects of this mutation on the disposition of drug binding residues in the pore below the selectivity filter (Hosaka et al., 2007) due to suppression of the hERG channel inactivation for this mutant (Mitcheson et al., 2000a; Melgari et al., 2015). T623, S624, G648, and Y652 are also known to affect flecainide inhibition of hERG to a lesser degree (Melgari et al., 2015). Moxifloxacin is a fluoroquinolone antibiotic drug that blocks hERG with reported IC_50_ values of ∼36–129 µM (Kang et al., 2001; Chen et al., 2005; Alexandrou et al., 2006; Qiu et al., 2016). Experimental data suggest that Y652 and F656 in the S6 segment and S624 at the base of the SF play key roles in the ability of moxifloxacin to block hERG channels (Alexandrou et al., 2006). Sotalol is both a class II (beta-adrenergic receptor-blocking) and class III (K^+^ channel-blocking) antiarrhythmic sulfonamide drug comprising d- and l-enantiomers (Funck-Brentano, 1993). Sotalol is a low-affinity binder to hERG with reported IC_50_ values of ∼290 µM (Dubois et al., 2016; Zhang et al., 2017; Ridder et al., 2020; DeMarco et al., 2021). Experimental data suggest that Y652 in the S6 segment plays important roles in the ability of sotalol to block hERG, but unlike high-affinity sulfonamide blockers, sotalol binding is not affected by SF residue S624 mutations. Dofetilide is a sulfonamide class III antiarrhythmic drug used in the treatment of ventricular arrhythmia. Dofetilide binds to the hERG channel in a state-dependent manner with a 70-fold higher affinity for an inactivated state (IC_50_ is ∼50–100 nM) and reported IC_50_ values of 3.5–11 µM for an open-state block (Lynch et al., 1995; Weerapura et al., 2002; Perrin et al., 2008; Vijayvergiya et al., 2015; Ridder et al., 2020). F656 is a molecular determinant of high-affinity binding and plays a key role in the ability of dofetilide to block hERG channels (Lees-Miller et al., 2000; Kamiya et al., 2006). Additionally, the pore helix (T623, S624, and V625) and S6 domain (G648, Y652, and V659) residues are known from alanine-scanning mutagenesis studies to reduce the block of methanesulfonanilide drugs such as dofetilide (Kamiya et al., 2006).

Our results reveal key similarities and differences between various drug interactions with wild-type and mutant hERG channels in open and closed states and provide useful structural insights into molecular mechanisms of drug action on the hERG current.

## 2 Materials and methods

### 2.1 Rosetta modeling of hERG in open and closed states

We used Rosetta structural modeling software (Bender et al., 2016; Leman et al., 2020) and the cryo-EM structures of a putatively open-state hERG channel (PDB ID: 5VA2) (Wang and MacKinnon, 2017) and closed-state EAG1 (PDB ID: 5K7L) (Whicher and MacKinnon, 2016) as templates to model hERG in open and closed states (Figure 1). Each structure was passed through the cryo-EM density refinement protocol in Rosetta (Wang et al., 2016) (Supplementary Script S1). The lowest-scoring density-refitted models were then used in RosettaCM (Song et al., 2013) to model the unresolved residues and atoms of the channel in the extracellular region (Supplementary Script S2). We generated 10,000 structural models of both open and closed states and selected the top 1,000 models from each for RosettaLigand modeling of hERG interaction with drugs (described as follows). The lowest-energy structures were visually inspected before being selected for the docking study. UCSF Chimera’s Rotamers tool was used to prepare the F656A, Y652A, and Y652A/F656A mutations based on the final wild-type open-state model. Coordinates of the top Rosetta score models of the hERG WT in closed and open states are provided in Supplementary Material.

### 2.2 RosettaLigand modeling of hERG interaction with drugs

We obtained the molecular structures of each drug from the ZINC (Irwin and Shoichet, 2005) and PubChem (Kim et al., 2019) databases. OpenEye OMEGA (OpenEye Scientific Software) was used to generate conformers for the drugs (Hawkins et al., 2010). At physiological pH 7.4, each drug exists in a dominant ionized (cationic or zwitterionic) form. However, because the drug receptor site in the pore lumen region is hydrophobic, this may shift the ionization equilibrium. This indicates that we need to study both ionized and neutral forms of each drug when analyzing its interactions with the hERG channel (Table 1) (Chatelain et al., 1986; Cross et al., 1990; Kodama et al., 1997; Hille, 2001; Lemaire et al., 2011; Kazusa et al., 2014). Previous computational studies suggest that the cationic form of sotalol predominantly remains in an aqueous solution, while the neutral form embeds into the membrane and interacts with the hERG channel (Yang et al., 2020; DeMarco et al., 2021), hence our inclusion of neutral drug docking results in the main text.

To uniformly and efficiently sample the pore region, drugs were placed at 10 different initial locations spanning the top and bottom of the pore lumen region and the four potential fenestration regions, formed by M554, F557, and A558 (on the S5 segment), L622 and T623 (on the P-helix), and L646, S649, L650, and A653 (on the S6 segment). As part of the standard Rosetta docking protocol, we set the initial random perturbation to a translation distance of less than 5 Å and the sampling radius to 5 Å (Supplementary Script S3). The details of the RosettaLigand docking algorithm have been described previously (Meiler and Baker, 2006; Davis and Baker, 2009; Lemmon and Meiler, 2012; Combs et al., 2013; Bender et al., 2016; Leman et al., 2020). A total of 100,000 docking models were generated for each drug and each protein. The top 10,000 were selected based on the total_score of the protein–ligand complex and then ranked by ligand binding energy represented by the Rosetta interface_delta_X score term. The top 50 most favorable interface score models were visually analyzed using UCSF Chimera (Pettersen et al., 2004). The representative poses were further analyzed using the Protein–Ligand Interaction Profiler (PLIP) (Salentin et al., 2015) web service. Coordinates of the top Rosetta score models of the hERG–drug complexes are provided in Supplementary Material. We uploaded the top 50 models for each hERG–drug complex presented in this study to the Dryad database (https://doi.org/10.5061/dryad.dfn2z357q).

Clustering analysis of the docking results was carried out in RStudio by calculating a similarity matrix between all top 50 poses clustered based on a cutoff parameter and minimum cluster size parameter using Eq. 1, where *z* is the drug center of mass (COM) position with respect to hERG SF C_α_ COM along the *z*-axis, *l* is the length of the vector between endpoint atoms of a drug molecule, and Φ is the polar angle of the drug endpoint atom vectors away from the *z*-axis (Supplementary Table S1 and Supplementary Scripts S4, S5).

$$Similarity=\sqrt{{\left(\frac{z-z_{ref}}{z_{\max}-z_{\min}}\right)}^{2}+{\left(\frac{l-l_{ref}}{l_{\max}-l_{\min}}\right)}^{2}+{\left(\frac{\Phi -\Phi_{ref}}{\Phi_{\max}-\Phi_{\min}}\right)}^{2}}.$$ (1)

This ensures invariance to the rotation around the *z*-axis and, therefore, can account for the 4-fold symmetry of the hERG channel. Unique clusters were then identified using K-means optimization initialized using the lowest interface score structures from each cluster as the cluster centers.

**FIGURE 2:**
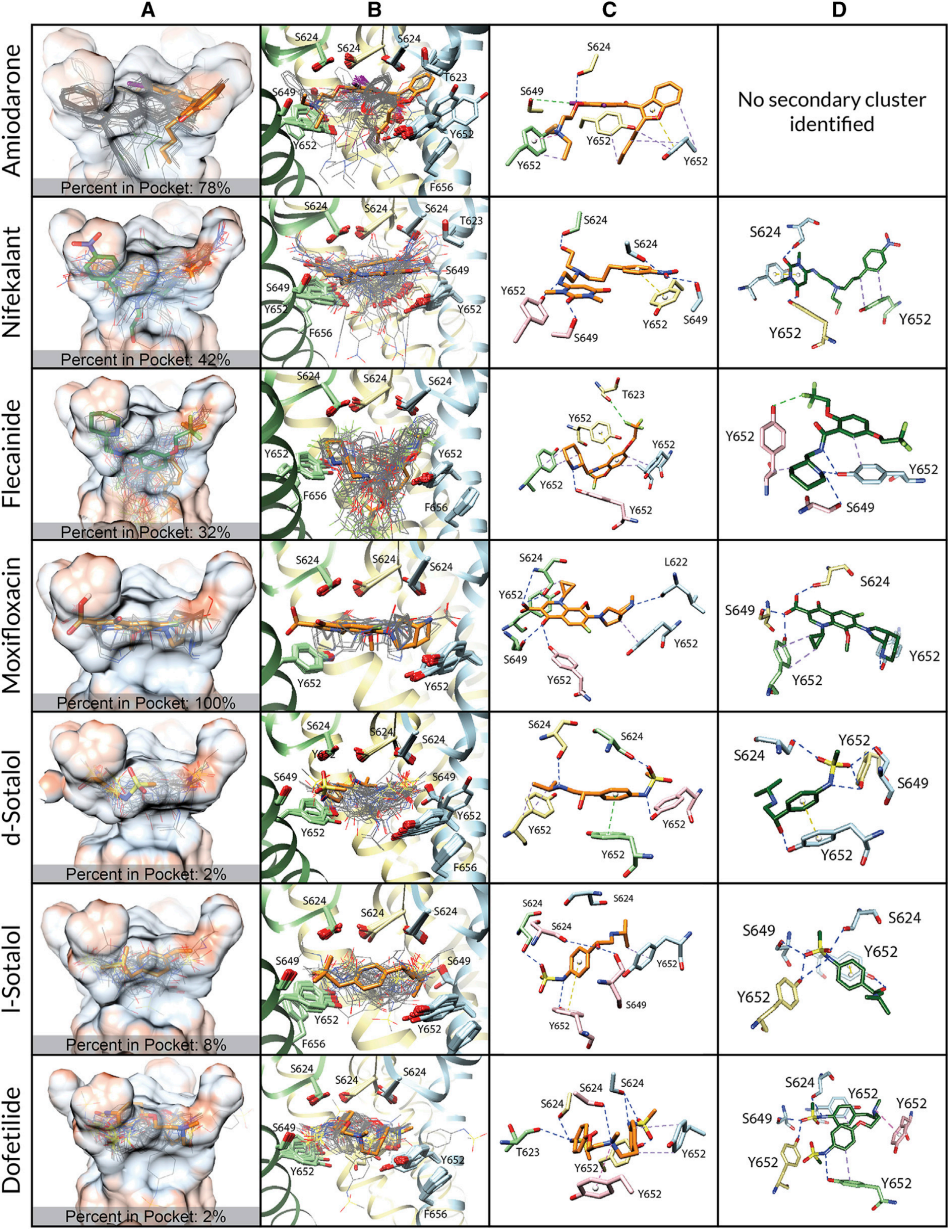
Docking of neutral drug forms to the WT hERG channel in an open state. **(A)** Rosetta docking results show the top 50 drug poses in wire representation and in gray. Models representing the largest and second-largest clusters are shown in stick representation with carbon atoms in orange and green, respectively. Other drug atoms are shown as follows: O atoms in red, N atoms in blue, S atoms in yellow, and I atoms in violet. hERG pore-lining residues are shown in surface representation and colored by hydrophobicity using UCSF Chimera: from blue for the most hydrophilic and white to red for the most hydrophobic. **(B)** Rosetta docking results with hERG channel pore-lining helices shown in ribbon representation and key residues shown in stick representation. Chain A is removed for clarity, chain B is in blue, chain C is in yellow, and chain D is in green. **(C and D)** Representative poses are identified as the lowest-energy pose from the largest (*panel c*) and second-largest (*panel d*) clusters, respectively. PLIP-identified interactions are indicated by dashed lines. Halogen bonds are in green, hydrogen bonds are in blue, cation–π interactions are in pink, π-stacking interactions are in yellow, and hydrophobic interactions are in pale purple.

The percentage within the hydrophobic pocket was calculated by the proportion of poses of the top 50 models of each docking simulation, with at least one atom positioned at or in the hydrophobic pocket of the hERG channel, as visualized for each pose in Chimera (Figure 2). The percentage within the closed pore of the hERG channel was calculated by counting the number of poses of the top 50 models for each docking simulation that are fully encapsulated within the hERG channel pore or fenestration region (Figure 1D).

### 2.3 IUPAC drug nomenclature

Amiodarone, (2-butyl-1-benzofuran-3-yl)-[4-[2-(diethylamino)ethoxy]-3,5-diiodophenyl]methanone.

Nifekalant, 6-[(2-((2-hydroxyethyl)[3-(4-nitrophenyl)propyl]amino)ethyl)amino]-1,3-dimethylpyrimidine-2,4(1H, 3H)-dione.

Flecainide, (RS)-N-(piperidin-2-ylmethyl)-2,5-bis(2,2,2-trifluoroethoxy)benzamide.

Moxifloxacin, 1-cyclopropyl-7-[(1S, 6S)-2,8-diazabicyclo [4.3.0]nonan-8-yl]-6-fluoro-8-methoxy-4-oxoquinoline-3-carboxylic acid.

Sotalol, (RS)-N-[4-[1-hydroxy-2-(propan-2-ylamino)ethyl]phenyl]methanesulfonamide.

Dofetilide, N-[4-(2-([2-(4-methane sulfonamidophenoxy)ethyl] (methyl)amino)ethyl)phenyl]methanesulfonamide.

## 3 Results and discussion

### 3.1 Rosetta modeling of the hERG channel in open and closed states

We used Rosetta structural modeling software (Bender et al., 2016; Leman et al., 2020) and cryo-EM structures of a putatively open-state hERG (Wang and MacKinnon, 2017) and closed-state EAG1 (Whicher and MacKinnon, 2016) channel (Figure 1A) as templates for generating open- and closed-state hERG models, respectively, as described previously. Comparison of the SFs of the open- and closed-state models to the original cryo-EM structures (Figure 1B) shows root-mean-square deviations (RMSDs) of 0.6 Å and 0.7 Å, respectively. There is an RMSD of 0.4 Å between the open- and closed-state SFs. HOLE (Smart et al., 1993; Smart et al., 1996) (Figure 1C) estimates the maximal pore radius of the open and closed hERG channel states in the putative drug-binding region to be ∼4.2 Å and ∼2.8 Å, respectively, with the greatest constriction of the pore due to the positioning of Y652 and F656. Cross-sections of the protein surface (Figure 1D) by Chimera were unable to detect any accessible pore lumen in the closed state of the hERG channel—although it could accommodate certain drugs (see Figure 6 and Supplementary Figure S5)—but revealed the wide pore lumen and deep hydrophobic pockets that extend up from the pore at the base of the open-state hERG SF (Figure 1D, right).

### 3.2 Modeling of drug interaction with the hERG channel using RosettaLigand

To study the binding of drugs with high (d/l-sotalol and dofetilide), intermediate (flecainide and moxifloxacin), and low (amiodarone and nifekalant) risks for arrhythmia (Table 2) to the hERG channel pore in the open and closed states, we used RosettaLigand, as described previously. PLIP analysis was used to identify hydrophobic interactions and hydrogen and halogen bonds, as well as π–π and cation–π interactions, in top-scoring Rosetta models. Drug-binding poses were clustered using a similarity matrix, which is based on drug conformation, pore *z* position, and orientation and implicitly accounts for a 4-fold symmetry of the hERG channel.

**TABLE 2:** Summary of drug docking top cluster size, interface energy (IE), key residues forming a receptor site, and specific pore region involved in binding for the WT and mutant hERG channels in an open state for each drug studied. In the *“Top Cluster Size and I.E.”* column, the cluster size, the number of poses in the cluster, is shown on the top, whereas IE in Rosetta Energy Unit (REU) is shown at the bottom.

Drug	Ion. St	WT - open state						Y652 A			F656 A			Y652A/F656 A		
		Top cluster Size and IE	Top cluster	Top cluster	Second cluster	Second cluster	Second cluster	Top cluster	Top cluster	Top cluster	Top cluster	Top cluster	Top cluster	Top cluster	Top cluster	Top cluster
			Key residues	Pore regions	Size and IE	Key residues	Pore regions	Size and IE	Key residues	Pore regions	Size and IE	Key residues	Pore regions	Size and IE	Key residues	Pore regions
Amiodarone	0	41	S624 S649 Y652	SF base hydrophobic pocket	n/a	n/a	n/a	28	F557, S649 A652, A653 I655, F656	Fenestration	36	S624 Y652	SF base hydrophobic pocket	45	S624 A653 A656	SF base hydrophobic pocket
		−19.2 REU						−15.9 REU			−18.3 REU			−15.2 REU		
	+1	27	L622, S624 V625, Y652	SF base hydrophobic pocket	16	S624 Y652	SF base hydrophobic pocket	31	S624 A652 F656	SF base hydrophobic pocket	23	S624 Y652	SF base hydrophobic pocket	30	S624 A652	SF base hydrophobic pocket
		−17.7 REU			−16.5 REU			−15.6 REU			−17.7 REU			−13.6 REU		
Nifekalant	0	13	S624 S649 Y652	SF base	6	S624 Y652	SF base hydrophobic pocket	8	F557, L622 S624, A652 I655, F656	SF base fenestration	17	L622, S624 S649, Y652	SF base	11 −15.5 REU	F557, S624 S649, A652	SF base fenestration
		−18.1 REU			−16.5 REU			−15.0 REU			−21.5 REU					
	+1	14	S649 Y652	SF base hydrophobic pocket	12	S624, S649 Y652, A653	SF base	24	S624 S649	SF base	26	S649 Y652	SF base hydrophobic pocket	11	F557 S624 A652	SF base fenestration
		−16.9 REU			−16.7 REU			−15.2 REU			−18.5 REU			−14.8 REU		
Flecainide	0	10	T623 Y652	SF base hydrophobic pocket	8	S649 Y652	Hydrophobic pcoket	22	S624, S649 A652, F656	SF base	9	Y652 A653	SF base hydrophobic pocket	28	S624 S649 A656	SF base
		−9.6 REU			−10.2 REU			−9.9 REU			−11.5 REU			−9.1 REU		
	+1	9	S624 S649 Y652	SF base hydrophobic pocket	6	L622 S649 Y652	Hydrophobic pocket	25	S624, S649 A653, F656	SF base	13	T623, S624 S649, Y652	SF base hydrophobic pocket	37	S624 A652 A656	SF base
		−11.1 REU			−10.8 REU			−12.1 REU			−9.9 REU			−9.8 REU		
Moxifloxacin	0	16	L622, S624 S649, Y652	SF base hydrophobic pocket	10	S624 S649 Y652	SF base hydrophobic pocket	21	S624, S649 A652, F656	SF base	14	L622, S624 S649, Y652	SF base hydrophobic pocket	12	S624 S649 A654	SF base
		−12.7 REU			−13.2 REU			−10.8 REU			−13.0 REU			−10.2 REU		
	Z	27	S624 S649 Y652	SF base hydrophobic pocket	8	S624, S649 Y652, A653	SF base hydrophobic pocket	26	S649, A653 Y652, S660	Central cavity only	21	S624 S649 Y652	SF base	37	T623, S624 G648, S649 A652	SF base hydrophobic pocket
		−13.3 REU			−12.5 REU			−11.9 REU			−13.2 REU			−10.5 REU		
d-Sotalol	0	14	S624 Y652	SF base	8	S624 S649 Y652	SF base	13	S624, S649 A652, F656	SF base	11	S624 Y652	SF base	10	T623, S624 S649, A652	SF base hydrophobic pocket
		−12.6 REU			−11.0 REU			−9.9 REU			−13.3 REU			−9.8 REU		
	+1	12	T623 S624 Y652	SF base	8	T623, S624 S649, Y652	SF base hydrophobic pocket	8	S624 S649	SF base	13	S624, V625 M645, S649 Y652	SF base hydrophobic pocket	14	S624 S649	SF base
		−13.0 REU			−13.0 REU			−10.1 REU			−13.3 REU			−9.9 REU		
l-Sotalol	0	9	S624 S649 Y652	SF base	5	S624 S649 Y652	SF base	13	T623, S624 A653, F656	SF base	14	T623 S624 Y652	SF base	27	S649 A652	Central cavity only
		−11.5 REU			−11.2 REU			−10.1 REU			−12.2 REU			−9.5 REU		
	+1	8	T623, S624 V625, Y652	SF base hydrophobic pocket	4	S624, S649 Y652, A653	SF base	7	S624, V625 A652, F656	Sf base hydrophobic pocket	15	T623, S624 V625, Y652	SF base hydrophobic pocket	37	S649 A652 A653	Central cavity only
		−12.0 REU			−12.3 REU			−11.2 REU			−13.9 REU			−10.2 REU		
Dofetilide	0	15	T623 S624 Y652	SF base	11	S624 S649 Y652	SF base	14	S624 A654 F656	SF base	9	S624 Y652	SF base	10	T623, S624 S649, A652	SF base
		−14.1 REU			−13.8 REU			−12.9 REU			−14.7 REU			−10.7 REU		
	+1	11	S624 Y652	SF base	11	S624 S649 Y652	SF base	19	T623, S624 S649, A652	SF base	11	T623 S624 Y652	SF base	11	L622 A652	SF base
		−13.4 REU			−13.1 REU			−11.3 REU			−13.9 REU			−10.5 REU		

### 3.3 Open-state WT hERG channel–drug interactions

We first studied open-state WT hERG interactions with neutral (Figure 2) and cationic or zwitterionic (Supplementary Figure S1) drugs. Every drug pose was initially positioned in the center of the pore, below the SF adjacent to Y652. Variabilities between drug poses are characterized by the frequency and depth of positioning within the hydrophobic pocket (Figure 2 and Supplementary Figures S1A, B; Table 2) and the variation in key binding residues of top clusters (Figure 2 and Supplementary Figures S1C, D; Table 2). Rosetta-predicted interface scores cannot be compared between different drugs. Notably, there are four deep hydrophobic pockets extending from the central pore cavity up behind the selectivity filter between the S6 helix and the pore helix and formed by residues T623, S624, V625, G648, and Y652, as identified in the cryo-EM hERG structure by Wang and MacKinnon (2017). These deep hydrophobic pockets are open and available to drugs in this putatively open-state WT hERG model and disappear in the closed-state WT hERG model (Figure 1D).

***Amiodarone.*** The most frequently sampled, lowest-binding energy RosettaLigand poses of both neutral (Figure 2) and cationic amiodarone (Supplementary Figure S1) interact with the WT open-state hERG channel pore in the region above F656 in the S6 segment and below the SF, with cationic amiodarone in an inverted orientation when compared to neutral amiodarone. For neutral amiodarone, 41 of the top 50 poses converged on a similar ligand orientation characterized by 1) the benzofuranyl group protruding up into the hydrophobic pocket; 2) iodine on the benzene ring accepting a hydrogen bond from S649 on the S6 segment; and 3) the butyl and ethyl groups at each end forming hydrophobic interactions with Y652 on opposing chains (Figure 2). For cationic amiodarone, 27 of the top 50 poses converged to a single cluster characterized by 1) the diethylammonium group protruding into the hydrophobic pocket; 2) iodine on the benzene ring forming a hydrogen bond with L622 on the pore helix near the base of the SF; 3) a π-stacking interaction between Y652 on one chain and the benzene ring of amiodarone; and 4) hydrophobic interactions between Y652 on multiple chains and the benzofuranyl and diethylammonium moieties of amiodarone (Supplementary Figure S1). A second, smaller cluster was identified for cationic amiodarone that resembles the top cluster of neutral amiodarone.

***Nifekalant.*** For neutral nifekalant, 13 of the top 50 RosettaLigand poses converged on a similar ligand orientation characterized by 1) π-stacking between the phenyl group and Y652 and 2) various hydrogen bonds between residues S624, S649, and Y652 and the pyrimidinedione, nitrophenyl, and hydroxyethylamino groups (Figure 2). A second, smaller cluster converged on a pose similar to the top cluster for cationic nifekalant, where the nitrophenyl group protruded further up into the hydrophobic pocket (Figure 2). For cationic nifekalant, 14 of the top 50 poses converged on a similar ligand orientation characterized by 1) the nitrophenyl group protruding up into the hydrophobic pocket; 2) hydrogen bonds between oxygen atoms of the nitrophenyl and pyrimidinedione moieties of nifekalant and S649 and Y652 residue sidechains on multiple chains; and 3) the hydrophobic interactions between Y652 and the drug’s phenyl group (Supplementary Figure S1). A second, smaller cluster converged on a pose where the pyrimidinedione moiety remained in the space between Y652 and S624, while the nitrophenyl group dipped down further into the pore rather than into the hydrophobic pocket.

***Flecainide.*** The most frequently sampled, lowest-binding energy RosettaLigand poses of both neutral (Figure 2) and cationic (Supplementary Figure S1) flecainide interact with the WT open-state hERG channel model pores in the region below the SF and extending down toward F656. For neutral flecainide, 10 of the top 50 poses converged on a similar ligand orientation characterized by 1) π-stacking between the drug’s benzene ring and Y652; 2) hydrogen bonding between Y652 and the drug’s piperidine group; 3) hydrophobic interactions between multiple Y652 residues and both the benzene and piperidine groups of flecainide; and 4) a hydrogen bond between T623 and a fluorine atom on the drug’s trifluoroethoxy group (Figure 2). A second, smaller cluster converged on a pose similar to the top cluster for cationic nifekalant where the piperidine group protruded further up into the hydrophobic pocket. For cationic flecainide, 9 of the top 50 poses also converged on a similar ligand orientation characterized by 1) the piperidine group protruding up into the hydrophobic pocket; 2) hydrogen bonds between S624, S649, and Y652 sidechain residues on multiple chains and oxygen atoms of the trifluoroethoxy and amide moieties and nitrogen of the piperidine group; 3) π–π stacking between Y652 and the benzamide group; and 4) a hydrogen bond between S649 and a fluorine atom on one trifluoroethoxy group (Supplementary Figure S1).

***Moxifloxacin.*** The most frequently sampled, lowest-binding energy RosettaLigand poses of neutral (Figure 1) and zwitterionic (Supplementary Figure S1) moxifloxacin interact with the WT open-state hERG channel pore in the region below the SF, while only some poses of the zwitterionic form extend down toward F656. For neutral moxifloxacin, 16 of the top 50 poses converged on a similar ligand orientation characterized by 1) hydrogen bonding between both sidechain atoms on L622, S624, S649, and Y652 and backbone atoms on S624 and various positions on moxifloxacin and 2) hydrophobic interactions between multiple Y652 residues and the cyclopropyl and pyrrolopyridine groups of moxifloxacin (Figure 2). Several smaller clusters similar to the largest cluster are positioned in the same region of the pore between the SF and Y652. For zwitterionic moxifloxacin, 27 of the top 50 poses also converged on a very similar ligand orientation characterized by hydrogen bonding between both sidechain S624, S649, and Y652 atoms and backbone S624 atoms with various groups of moxifloxacin and 2) hydrophobic interactions between multiple Y652 residues and the cyclopropyl and pyrrolopyridine groups of moxifloxacin (Supplementary Figure S1). Thirteen of the top 50 poses of zwitterionic moxifloxacin extend into the fenestration region.

***Sotalol.*** In the most frequently sampled, lowest-binding energy RosettaLigand poses of neutral (Figure 2) and cationic (Supplementary Figure S1) d- and l-sotalol, both interact with the WT open-state hERG channel pore in the region below the SF and above Y652. There is little convergence of sotalol poses in the pore lumen for both WT and mutant poses, most likely due to sotalol being a low-affinity hERG binder (Dubois et al., 2016; Zhang et al., 2017; Ridder et al., 2020; DeMarco et al., 2021). Neutral d- and l-sotalol poses (Figure 2) remained centered in the open WT pore, forming hydrogen bonds and hydrophobic interactions with S624 and Y652 on multiple chains as well as a π–π stacking interaction between Y652 and the phenyl group of neutral sotalol. The 2-propylammonium group of the cationic l-sotalol top cluster and a secondary cluster of cationic d-sotalol (Supplementary Figure S1) reaches into the hydrophobic pocket interacting with T623 and V625. For neutral and cationic l-sotalol, the 2-propylamine group also formed hydrophobic interactions with T623 and V625. Two of the top 50 poses of cationic l-sotalol extend into the fenestration region.

***Dofetilide.*** The most frequently sampled, lowest-binding energy RosettaLigand poses of neutral (Figure 2) and cationic (Supplementary Figure S1) dofetilide both interact with the WT open-state hERG channel pore in the region above Y652 and below the SF. For neutral dofetilide, 15 of the top 50 poses converged on a similar ligand orientation characterized by 1) cation–π interactions between the central methylamine of dofetilide and Y652 and 2) various hydrogen bonds between residues T623 and S624 of multiple chains and the oxygen of the phenoxy and the nitrogen of the sulfonamide moieties and the central methylamine (Figure 2). Residues T623 and S624 affect high-affinity binding of drugs (Kamiya et al., 2006; Butler et al., 2019). Four additional clusters converged in the exact same region of the pore and in similar orientations. For cationic dofetilide, 11 of the top 50 poses converged on a very similar orientation as the top cluster for neutral dofetilide, along with several other identified clusters (Supplementary Figure S1). One cluster of cationic dofetilide did have a sulfonamide group protruding into the hydrophobic pocket, while 2 of the top 50 poses extended into the fenestration region.

***Comparison.*** Top clusters of amiodarone, nifekalant, and flecainide are frequently (32%–78% of poses) and deeply positioned within the hydrophobic pocket. All moxifloxacin poses are positioned at the opening of the hydrophobic pocket, while very few poses (2%–8%) of d/l-sotalol and dofetilide are positioned in the hydrophobic pocket. We hypothesize that drug binding within the hydrophobic pocket may allosterically affect channel gating by affecting the closure of the S6 helix bundle. This pattern of interaction with the hydrophobic pocket is consistent with experimental data, suggesting that facilitating drugs may act as a wedge to bias hERG channel equilibrium toward the open state and increase hERG current amplitude in response to low-voltage depolarization (Hosaka et al., 2007; Yamakawa et al., 2012; Furutani et al., 2019).

### 3.4 Open-state mutant hERG channel–drug interactions

Residues Y652 and F656 on the pore-lining S6 helix are known key determinants of drug binding in the hERG channel pore (Figure 1). Mutations of these residues would be expected to decrease binding affinity for each ligand. Rosetta interface scores (Table 2) are ligand-specific and cannot be compared across different drugs (Lemmon and Meiler, 2012) but can be compared across various protein mutants and conformational states.

**FIGURE 3:**
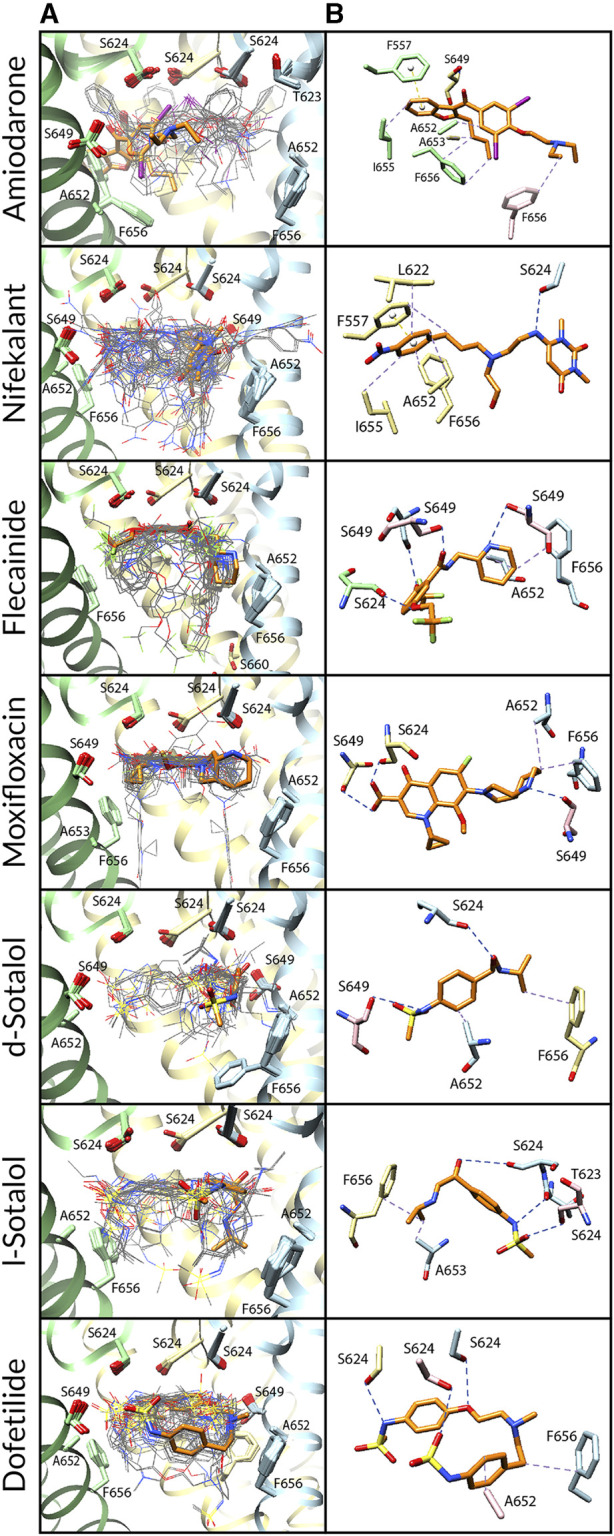
Docking of neutral drug forms to the Y652A hERG channel in an open state. **(A)** Rosetta docking results show the top 50 drug poses in wire representation and in gray. The top pose from the largest cluster is shown in stick representation and is orange. hERG channel chains and drug atoms are colored as shown in Figure 2. **(B)** Representative poses are identified as the lowest-energy pose from the largest cluster. PLIP-identified interactions are indicated and colored as shown in Figure 2.

**FIGURE 4:**
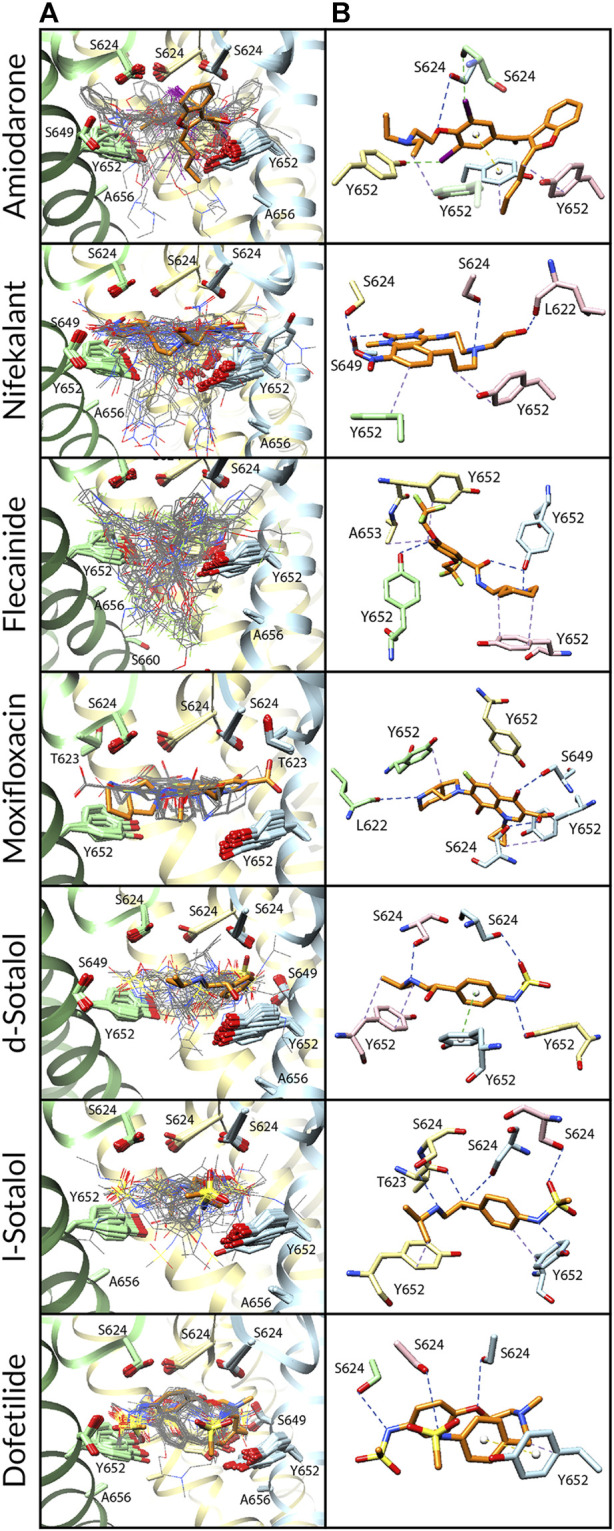
Docking of neutral drug forms to the F656A hERG channel in an open state. **(A)** Rosetta docking results show the top 50 drug poses in wire representation in gray. The top pose from the largest cluster is shown in stick representation in orange. hERG channel chains and drug atoms are colored as shown in Figure 2. **(B)** Representative poses are identified as the lowest-energy pose from the largest cluster. PLIP-identified interactions are indicated and colored as shown in Figure 2.

**FIGURE 5:**
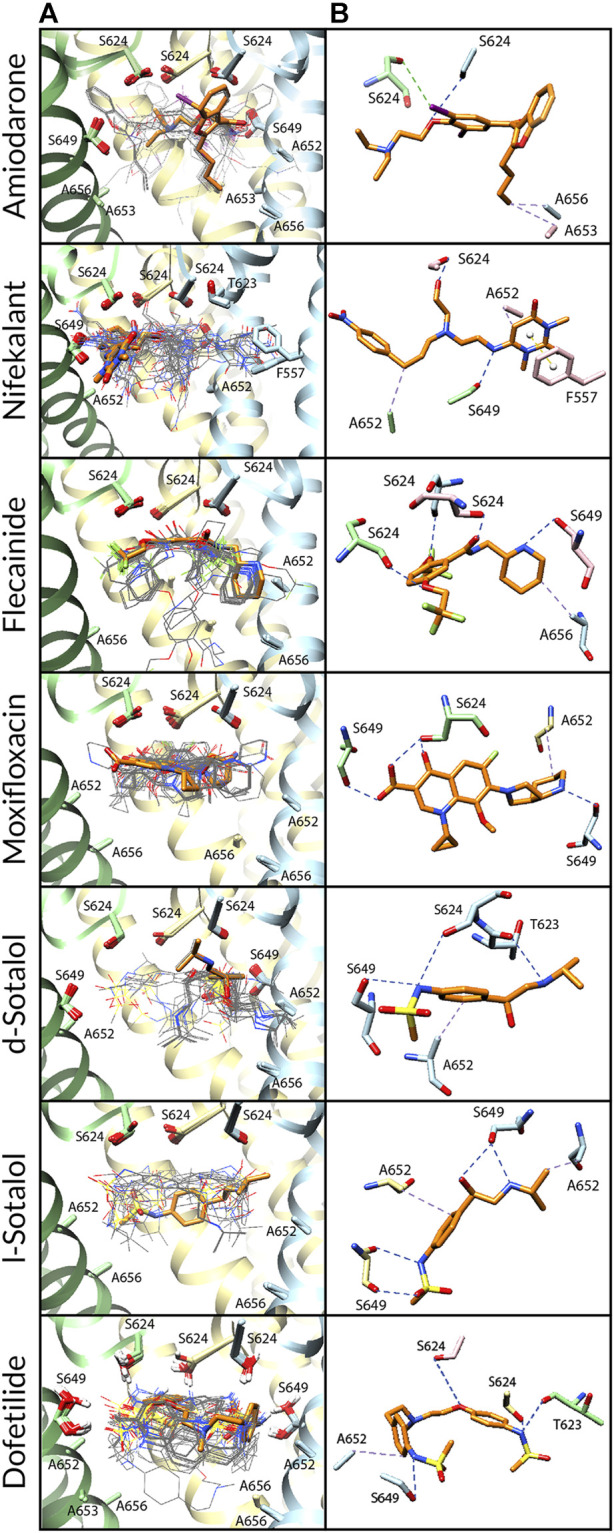
Docking of neutral drug forms to the Y652A/F656A hERG channel in an open state. **(A)** Rosetta docking results show the top 50 drug poses in wire representation in gray. The top pose from the largest cluster is shown in stick representation in orange. hERG channel chains and drug atoms are colored as shown in Figure 2. **(B)** Representative poses are identified as the lowest-energy pose from the largest cluster. PLIP-identified interactions are indicated and colored as shown in Figure 2.

***Amiodarone.*** Neutral and cationic amiodarone remained in a similar region within the hERG pore located between F656 and the base of the SF for all the hERG channel mutants (Figures 3–5). For neutral amiodarone complexes with the hERG F656A and Y652A/F656A mutants, the top cluster poses revealed the benzofuranyl group protruding into the hydrophobic pocket with the butyl and diethylamino groups forming various hydrophobic contacts with Y652, F656, and A653 (Figures 4, 5). The top cluster pose for neutral amiodarone docked to the hERG Y652A mutant showed the benzofuranyl group protruding into the fenestration region, forming a π-stacking interaction with F557 on the S5 helix and a hydrophobic interaction with I655 on the S6 helix (Figure 3). While the representative neutral amiodarone-binding pose for the F656A hERG channel mutant is not located low enough in the pore to interact with the mutated residue A656, the top clusters of neutral amiodarone poses docked to Y652A and double-mutant hERG channel models both form hydrophobic contacts lower in the pore with residue 656. For cationic amiodarone complexes with all the hERG mutants, the top cluster poses revealed the benzofuranyl group to be protruding into the hydrophobic pocket but a reduced number of predicted nonbonded interactions with the other functional groups of cationic amiodarone forming hydrophobic interactions with Y652 or A652 (Supplementary Figures S2-S4). The top cationic amiodarone pose of the top cluster only dips low enough into the pore to form hydrophobic interactions with F656 in the Y652A mutant hERG channel model. For F656A and Y652A/F656A but not Y652A mutants, iodine on the benzene ring is available to form halogen bonds with multiple S624 residues at the base of the SF. However, for the Y652A mutant, the oxygen in the ethoxy group connected to the benzene ring does create a hydrogen bond with the hydroxyl group of S624. For the F656A mutant interaction with both neutral and cationic amiodarone and the Y652A mutant with neutral amiodarone, the largest low-energy clusters are smaller in size compared to the WT, suggesting less convergence. The largest low-energy cluster of the Y652A hERG mutant interaction with cationic amiodarone and the double-mutant clusters with both neutral and cationic amiodarone are larger than those for the WT, showing higher convergence.

***Nifekalant.*** Neutral (Figures 3–5) and cationic (Supplementary Figures S2-S4) nifekalant remained in the region within the hERG pore located between residue 656 and the base of the SF for all the hERG channel mutants. However, both neutral and cationic nifekalant showed a reduced number of poses interacting with the hydrophobic pocket for all hERG channel mutants. For neutral nifekalant complexes with the Y652A and F656A/Y652A mutants as well as cationic nifekalant with the F656A/Y652A mutant, the largest clusters protrude into the fenestration region facing the lipid membrane and forming π-stacking interactions with F557 on the S5 helix.

***Flecainide.*** Neutral (Figures 3–5) and cationic (Supplementary Figures S2-S4) flecainide clusters remained in the region within the hERG pore located between Y656 and the base of the SF for all the hERG channel mutants, although some poses extended further down to interact with S660. However, both neutral and cationic flecainide showed a reduced number of poses interacting with the hydrophobic pocket for Y652A and F656A/Y652A hERG mutants, while F656A showed more similarity to WT. For neutral and cationic flecainide complexes with the Y652A and F656A/Y652A mutants, the largest clusters do not show any π-stacking, and almost no poses extend into the hydrophobic pocket or fenestration regions.

***Moxifloxacin.*** Neutral moxifloxacin (Figures 3–5) clusters remained tightly clustered in the region within the hERG pore located between residue 652 and the base of the SF for all the hERG channel mutants in a similar pose to the open-state WT, although some poses extended further down to interact with residues 656 and S660 (Figure 3). Zwitterionic moxifloxacin (Supplementary Figures S2-S4), in contrast, did not remain as tightly clustered. For the Y652A mutant, 26 of the top 50 poses cluster with the dihydroquinoline group of zwitterionic moxifloxacin extending down into the pore toward A656 (Figure 3). For the F656A mutant, 40 of the top 50 poses of zwitterionic moxifloxacin remain in the region between the SF and Y652, while 10 poses have the diazabicyclononanyl group tilted down below the Y652 toward A656 (Figure 4). For the Y652A/F656A double mutant, zwitterionic moxifloxacin was tightly clustered with 37 of the top 50 poses remaining in the pore region between the bottom of the SF and A652 (Figure 5). The only interactions with the hydrophobic pocket were for zwitterionic moxifloxacin with the Y652A/F656A double mutant. However, several moxifloxacin poses extend toward the fenestration region in all open-state models further than dofetilide but do not reach out of the pore like nifekalant or amiodarone.

***Sotalol.*** The F656A mutation in the hERG channel model did not change the interaction profile for any form of sotalol (Figures 3–5) except that the cationic d-sotalol pose reaching into the hydrophobic pocket became the largest cluster rather than a secondary cluster. However, the Y652A and Y652A/F656A mutations distinctly change the interaction profiles for sotalol where the most frequently sampled, lowest-binding energy RosettaLigand poses of neutral and cationic d- and l-sotalol either shift inward to form hydrogen bonds with S649 or down to form hydrophobic interactions with F656. For the Y652A/F656A double mutant, the 2-propylamine group of neutral d-sotalol in the large secondary cluster extended out into the fenestration region. For neutral and cationic l-sotalol, the 2-propylamine group also interacted with the hydrophobic pocket of the Y652A mutant and formed hydrophobic interactions with T623 and V625.

***Dofetilide.*** Binding poses of neutral (Figures 3–5) and cationic (Supplementary Figures S2-S4) dofetilide interacting with hERG Y652A, F656A, and Y652A/F656A mutants in an open state remained in the region within the hERG pore located between Y652 and the base of the SF for all the hERG channel mutants as cluster size remained similar and no poses extended into the hydrophobic pockets or fenestration regions.

***Comparison.*** Open-state WT hERG interface scores are lower than, or similar to, those of Y652A mutants, suggesting that these poses are relevant for amiodarone, nifekalant, flecainide, moxifloxacin, d-sotalol, and dofetilide, based on the comparison to existing experimental data. Our structural modeling is consistent with experimental data showing less-potent IC_50_ values for the Y652A hERG mutant (Mitcheson et al., 2000a; Kamiya et al., 2006; Melgari et al., 2015; Zhang et al., 2016) and with previous drug–hERG computational docking studies showing that the Y652 sidechain forms a drug-binding site (Durdagi et al., 2012; Negami et al., 2019; Kudaibergenova et al., 2020; DeMarco et al., 2021). However, we are not able to accurately compare between ours and previously published drug poses from other studies because of the unavailability of their structural coordinates. In disagreement with experimental data, Rosetta interface scores for nifekalant, neutral flecainide, neutral moxifloxacin, d-sotalol, neutral l-sotalol, and dofetilide interacting with the the F656A mutant hERG model were more favorable than those with the open-state WT hERG model. This may be due to our study being limited to two conformational states based on available hERG and EAG1 cryo-EM structures and the inability of the RosettaDock method to sample local and allosteric conformational changes upon drug binding within and near the receptor site formed by F656. Furthermore, the RosettaLigand score function does not explicitly evaluate cation–π and *π*–π nonbonded interactions (Meiler and Baker, 2006; Combs et al., 2018), which may lead to inaccuracies in RosettaLigand docking scores and binding pose predictions. Notably, previous computational docking studies based on the hERG cryo-EM structure also did not identify F656 as a part of the drug receptor site (Helliwell et al., 2018; Vaz et al., 2018; Munawar et al., 2019; Kalyaanamoorthy et al., 2020). Specifically, a small rotation of the S6 segment from its conformation in the hERG cryo-EM structure was suggested to position F656 sidechains toward a drug-accessible receptor site (Helliwell et al., 2018; Kalyaanamoorthy et al., 2020). Indeed, molecular dynamics simulations revealed that conformational dynamics of F656 sidechains may contribute to state-dependent drug binding (Perissinotti et al., 2019; Dickson et al., 2020; Kalyaanamoorthy et al., 2020; Kudaibergenova et al., 2020; Yang et al., 2020; DeMarco et al., 2021; Koulgi et al., 2022; Das et al., 2023). Similarly, F656A and Y652A mutations can dramatically change hERG channel pore conformational preferences, which was not directly tested in our RosettaLigand docking calculations but can be probed by molecular dynamics simulations in our follow-up study. Our putatively open-state hERG channel model represents only one state out of other potentially open, inactivated, or intermediate states for which drugs may have a higher affinity (Wu et al., 2015) but which might be revealed only upon drug binding.

### 3.5 Closed-state WT hERG channel–drug interactions

The closed hERG channel pore can accommodate ligands of various sizes (Mitcheson et al., 2000a; Windisch et al., 2011; Linder et al., 2016; Munawar et al., 2019). Gating-induced changes in the spatial location of F656, as well as open-state interactions with key residues in the pore (S624, Y652, and F656), are considered to be particularly important in drug-trapping phenomena (Milnes et al., 2003; Witchel et al., 2004; Kamiya et al., 2006; Munawar et al., 2019). Experimental data suggest that flecainide, sotalol, and dofetilide can be trapped within the hERG channel pore as the channel gate closes, while amiodarone and moxifloxacin do not remain within the closed pore (Mitcheson et al., 2000b; Paul et al., 2002; Milnes et al., 2003; Perry et al., 2004; Witchel et al., 2004; Kamiya et al., 2006; Stork et al., 2007; Windisch et al., 2011; Linder et al., 2016; Munawar et al., 2019; Gomis-Tena et al., 2020).

**FIGURE 6:**
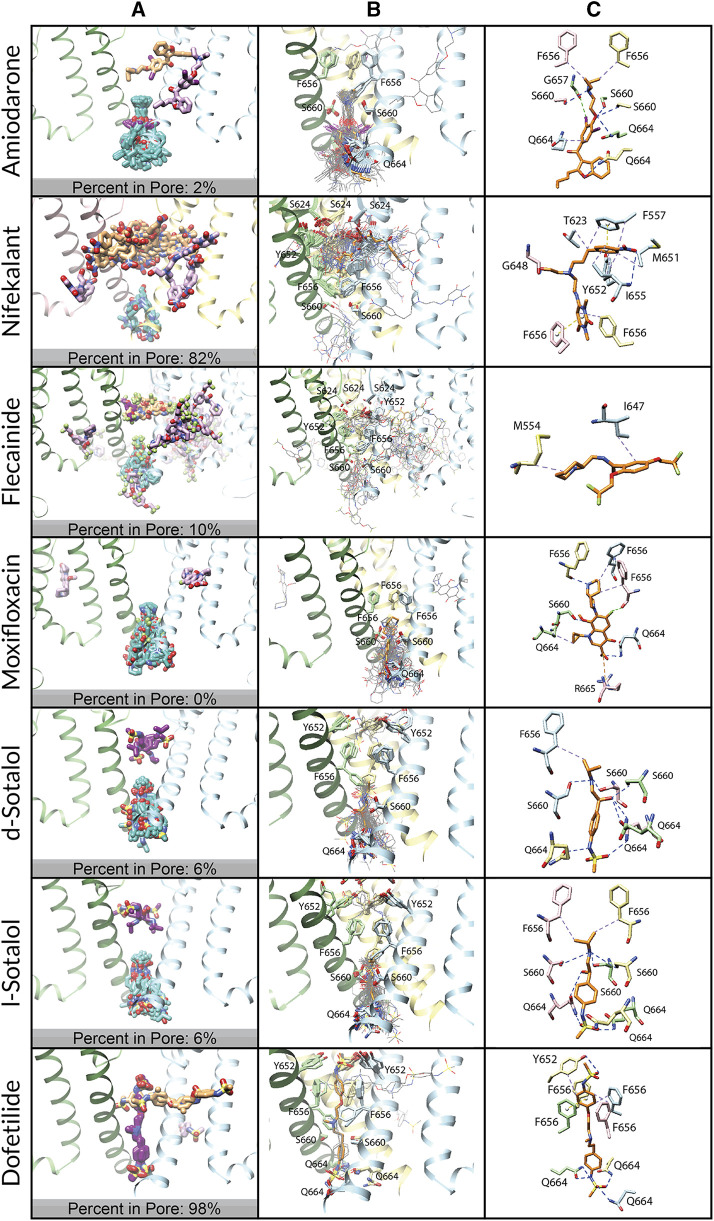
Docking of neutral drug forms to the WT hERG channel in a closed state. **(A)** Top 50 drug poses are colored by position relative to the hERG channel region: within the pore lumen is in dark magenta, within the fenestration is orange, within the intracellular gate is in cyan, and inside the membrane is in pale pink. hERG channel chains and drug atoms are colored as shown in Figure 2. **(B)** Rosetta docking results show the top 50 drug poses in wire representation in gray. The top pose from the largest cluster is shown in stick representation in orange. hERG channel chains and drug atoms are colored as shown in Figure 2. **(C)** Representative poses are identified as the lowest-energy pose from the largest cluster. PLIP-identified interactions are indicated and colored as shown in Figure 2.

***Amiodarone.*** Modeling of neutral (Figure 6) and cationic (Supplementary Figure S5) amiodarone to the closed hERG channel pore revealed that almost all top 50 binding poses for both neutral and cationic amiodarone can be characterized by 1) hydrophobic interactions between F656 and the diethylamino tail of amiodarone; 2) several hydrogen bonds between amiodarone oxygen and iodine atoms and G657, S660, and Q664 sidechains; and 3) the benzofuranyl group located at the base of the channel pore and extending into the intracellular region. For both neutral and cationic amiodarone binding to a closed-state model of the hERG channel, the interface scores of the representative poses were less favorable than for open-state WT but more favorable than for the double-mutant hERG channel models.

***Nifekalant.*** Modeling of neutral nifekalant (Figure 6) to the closed hERG channel pore revealed that 39 of the top 50 binding poses either extend into the fenestration region or remain in the pore with some protruding into the hydrophobic pocket. Of these, 11 of the top poses in a cluster converged to a similar ligand orientation characterized by 1) a π-stacking interaction between the nitrophenyl group and F557 on the S5 helix within the fenestration region; 2) π-stacking and hydrophobic interactions between the pyrimidinedione group and F656 within the pore; and 3) a hydrophobic interaction network between the nitrophenyl group and residues F557, T623, M651, and I655. In contrast, in the top 50 binding poses for cationic nifekalant (Supplementary Figure S5), almost all of them escape from the pore laterally toward the membrane or, in the largest cluster, shift down toward the intracellular side. Characterizing 27 of the top 50 poses that compose this largest cluster and converge on a similar ligand orientation are 1) parallel and perpendicular π-stacking interactions between Y652 and the nitrophenyl group; 2) hydrogen bonds between the pyrimidinedione and hydroxyethyl groups and residues S660 and Q664 on multiple chains; and 3) hydrophobic interactions between the nitrophenyl group and residues Y652 and F656.

***Flecainide.*** Modeling of neutral flecainide (Figure 6) to the closed hERG channel pore revealed that all top 50 binding poses either escape from the pore laterally toward the membrane or shift down to the intracellular gate. Similarly, in the top 50 binding poses for cationic flecainide (Supplementary Figure S5), all of them escape from the pore laterally toward the membrane or, in the second cluster, shift down toward the intracellular side. However, for cationic flecainide, two clusters can be clearly identified in these two areas with the top pose outside but near the fenestration region characterized by 1) a halogen bond between a trifluoroethoxy group and the backbone nitrogen of M645 and 2) a hydrophobic interaction network between the benzamide and piperidine groups and residues F551, A558, F619, I642, and L646.

***Moxifloxacin.*** Modeling of neutral (Figure 6) and zwitterionic (Supplementary Figure S5) moxifloxacin to the closed hERG channel pore revealed that nearly all top 50 binding poses shift down to the intracellular gate, and the largest clusters are characterized by 1) a halogen bond between the fluorine of moxifloxacin and the backbone oxygen of F656; 2) a hydrophobic interaction network between moxifloxacin and F656 and Q664 on multiple subunits; 3) a hydrogen bond network between moxifloxacin and S660, Q664, and the backbone of F656; and 4) a salt bridge between the carboxylic acid on moxifloxacin and R665.

***Sotalol.*** Modeling of neutral (Figure 6) and cationic (Supplementary Figure S5) d- and l-sotalol revealed that all of the top 50 binding poses for cationic and 94% of the top 50 poses for neutral d- and l-sotalol shift down to the intracellular gate. For neutral d- and l-sotalol, however, 6% of poses remain trapped in the pore interacting with Y652 and F656. All of the top cluster poses for neutral and cationic d- and l-sotalol can be characterized by large hydrogen bond networks between sotalol and S660 and Q664 residues of all four chains simultaneously, along with some hydrophobic interactions with the β-carbon of F656.

***Dofetilide.*** Modeling of neutral dofetilide (Figure 6) to the closed hERG channel pore revealed that 44 of the top 50 binding poses remained seemingly trapped in the pore region, with one sulfonamide end near the top of the pore below the SF and the other at the base of the pore, forming bonds with Y652 and Q664, respectively. This cluster converged to an orientation characterized by 1) a π-stacking interaction between the phenoxy group and F656; 2) hydrophobic interactions between residues Y652 and F656 and the phenoxy groups; and 3) hydrogen bonds between residues Y652 and Q664 on multiple chains and both sulfonamide groups. Similarly, the top cluster for cationic dofetilide (Supplementary Figure S5) also remains trapped within the closed state in an extended conformation within the pore. This cluster, however, is only composed of nine models and is shifted to sit higher within the pore with one sulfonamide end protruding into the fenestration region while the other reaches down to S660. The remaining 41 of the top 50 binding poses for cationic dofetilide escape from the pore either laterally toward the membrane or shift down toward the intracellular gate.

***Comparison.*** Either none or one of the top 50 docked drug poses remains in the pore for moxifloxacin or amiodarone, respectively, while many of the top 50 docked poses (6%–98%) remain within the pore cavity for sotalol, flecainide, nifekalant, and dofetilide. These interactions with the closed state may reflect experimentally observed trapped behavior. While all sotalol and all but one top docked poses of dofetilide are entirely contained within the pore cavity, the docked poses remaining within the pore for nifekalant and flecainide prefer the fenestration region, where one end of the ligand is within the pore cavity while the other end extends horizontally from the pore toward the cell membrane. The percentage of drug poses remaining within the closed-state hERG channels suggests that they can accommodate known trapped drugs (flecainide, d/l-sotalol, and dofetilide) but not non-trapped drugs (amiodarone or moxifloxacin). Trapped drugs that do not demonstrate facilitation effects (sotalol and dofetilide) do not interact with the closed-state pore regions expected to affect channel gating. Our models predict that nifekalant may demonstrate trapping behavior.

## 4 Conclusion

We investigated similarities and differences between various drug interactions with the hERG channel in open and closed states and highlighted key structural insights into molecular mechanisms of drug action. RosettaLigand docking simulations using models of both the putatively open- and closed-state hERG channels suggest that previously identified residues (S624 and Y652) are important in binding for each drug studied, while other pore-lining residues (L622, T623, V625, and S649) are often also involved. These results are consistent with those of previous mutational studies and pharmacophore models which, together, identify hydrophobic features of drugs that may interact in/near the hydrophobic pocket (L622, T623, and V625), while ionizable functional groups may favor interactions on the S6 helix near S649 and Y652 (Cavalli et al., 2002; Milnes et al., 2003; Ridley et al., 2004; Clarke et al., 2006; Hosaka et al., 2007; Yamakawa et al., 2012).

Notably, top clusters of amiodarone, nifekalant, flecainide, and moxifloxacin positioned within the hydrophobic pocket, while few poses of d/l-sotalol and dofetilide are positioned in the hydrophobic pocket. We predict that drug interactions within the hydrophobic pocket—a region absent in our closed-state hERG model—may impact the closure of the S6 helix bundle, thereby affecting channel gating. Consistent with our results, experimental data suggest that facilitating drugs may act as a wedge to bias hERG channel equilibrium toward an open-state conformation and can increase hERG current amplitude in response to low-voltage depolarization (Hosaka et al., 2007; Yamakawa et al., 2012; Furutani et al., 2019).

More favorable interface energies of drug binding with the F656A hERG mutant than those of the WT hERG channel do not support experimental evidence of F656 as a key binding determinant for nifekalant, neutral flecainide, neutral moxifloxacin, d-sotalol, l-sotalol, and dofetilide. This may be due to the limitations of the RosettaDock approach to sample allosteric conformational changes upon drug binding near the receptor site formed by F656 and evaluate entropic contributions of F656 to ligand binding (Mobley and Dill, 2009). Furthermore, our putatively open-state hERG channel model represents only one state out of other potentially multiple putative open, inactivated, and intermediate states for which drugs may have a higher affinity but which are only available upon drug binding.

Our results suggest a potential structural model for hERG channel facilitation through drug interactions with the hydrophobic pocket region of the hERG pore domain. In our open-state hERG channel, models facilitating drugs interact with the hydrophobic pocket more than non-facilitating drugs. Furthermore, closed-channel pore-trapped, facilitating drugs interact with the hERG fenestration region more than trapped, non-facilitating drugs.

We consider our RosettaLigand method-based computational docking simulations of hERG–drug interactions to be complementary to previous multiple computational molecular docking and MD simulation studies (Durdagi et al., 2012; Helliwell et al., 2018; Vaz et al., 2018; Munawar et al., 2019; Negami et al., 2019; Perissinotti et al., 2019; Dickson et al., 2020; Kalyaanamoorthy et al., 2020; Kudaibergenova et al., 2020; Yang et al., 2020; DeMarco et al., 2021; Koulgi et al., 2022; Das et al., 2023). Experimental measurements and molecular dynamics simulations are needed to test computational docking-based structural hypotheses. New cryo-EM structures in multiple conformations representing open, closed, and inactivated hERG channel states in the presence and absence of ligands are needed to gain a better general understanding of drug binding to the hERG channel.

## Data Availability

The datasets presented in this study can be found in online repositories. The names of the repository/repositories and accession number(s) can be found in the article/Supplementary Material.

## Abbreviations

CiPA, Comprehensive *in vitro* Proarrhythmia Assay; COM, center of mass; cryo-EM, cryo-electron microscopy; EAG, ether-à-go-go; hERG, human ether-à-go-go-related gene; ICH, International Council for Harmonization of Technical Requirements for Pharmaceuticals for Human Use; IUPAC, International Union of Pure and Applied Chemistry; Kv, voltage-gated K^+^ channel; LQTS, long QT syndrome; PDB, Protein Data Bank; PD, pore-forming domain; PLIP, Protein–Ligand Interaction Server; SF, selectivity filter; TdP, torsade de pointes; VSD, voltage-sensing domain; WT, wild type.
