# Supplementary material for: Structural modeling of hERG channel–drug interactions using Rosetta
Source: Front Pharmacol. 2023 Nov 14;14:1244166. doi: 10.3389/fphar.2023.1244166 (PMC10682396; doi:10.3389/fphar.2023.1244166)
Supplement: Supplementary file 1 [file DataSheet1.pdf]

Supplementary Material for:

Structural Modeling of hERG Channel – Drug Interactions Using Rosetta

Aiyana M. Emigh Cortez[1,2], Kevin R. DeMarco[1,2], Kazuharu Furutani[2,3], Slava Bekker[2,4], Jon T. Sack[2,5], Heike Wulff[6], Colleen E. Clancy[2,6,7], Igor Vorobyov[2,6,*], Vladimir Yarov-Yarovoy[2,5,*]

[1]Biophysics Graduate Group, University of California Davis, CA, USA

[2]Department of Physiology and Membrane Biology, University of California Davis, CA, USA

[3]Department of Pharmacology, Tokushima Bunri University, Tokushima, Japan

[4]American River College, Sacramento, CA, USA

[5]Department of Anesthesiology and Pain Medicine, University of California Davis, CA, USA

[6]Department of Pharmacology, University of California Davis, CA, USA

[7]Center for Precision Medicine and Data Sciences, University of California Davis, CA, USA

*For correspondence:

yarovoy@ucdavis.edu (VY-Y)

ivorobyov@ucdavis.edu (IV)

Supplement Table 1. Clustering Parameters

Drug	Similarity Cutoff	Minimum Cluster Size	Endpoint Atoms
Amiodarone	0.1	3	C2, C22
Dofetilide	0.1	3	S1, S2
Flecainide	0.05	3*	C9, C16
Moxifloxacin	0.01	3	C11, O3
Nifekalant	0.1	3	O2, O3
d-Sotalol	0.05	3	C8, C9
l-Sotalol	0.05	3	C8, C9

*Neutral flecainide docked to closed-state hERG channel used a minimum cluster size of 2.

Supplement Figure 1. WT Open-State hERG Channel Docking with Cationic or Zwitterionic Drugs. Rosetta docking results of wild-type (WT) hERG in open state with cationic or zwitterionic forms of each drug. Chain A is colored pink, chain B is blue, chain C is yellow, chain D is green, and ligand C atoms are gray. Top pose from largest and second largest clusters are in stick form and are orange and dark green, respectively. O atoms – red, N – blue, S – yellow, I – violet. H atoms not shown for clarity. Panel A shows the surface of pore-lining residues colored by hydrophobicity. Panels A and B show top 50 poses in gray sorted by lowest interface score. Panels C and D highlight representative poses identified as the lowest energy pose from the largest and second largest cluster, respectively. PLIP-identified interactions are indicated by dashed lines. Halogen bonds are colored green, hydrogen bonds in blue, cation-pi in pink, pi-stacking in yellow, hydrophobic interactions in pale purple.

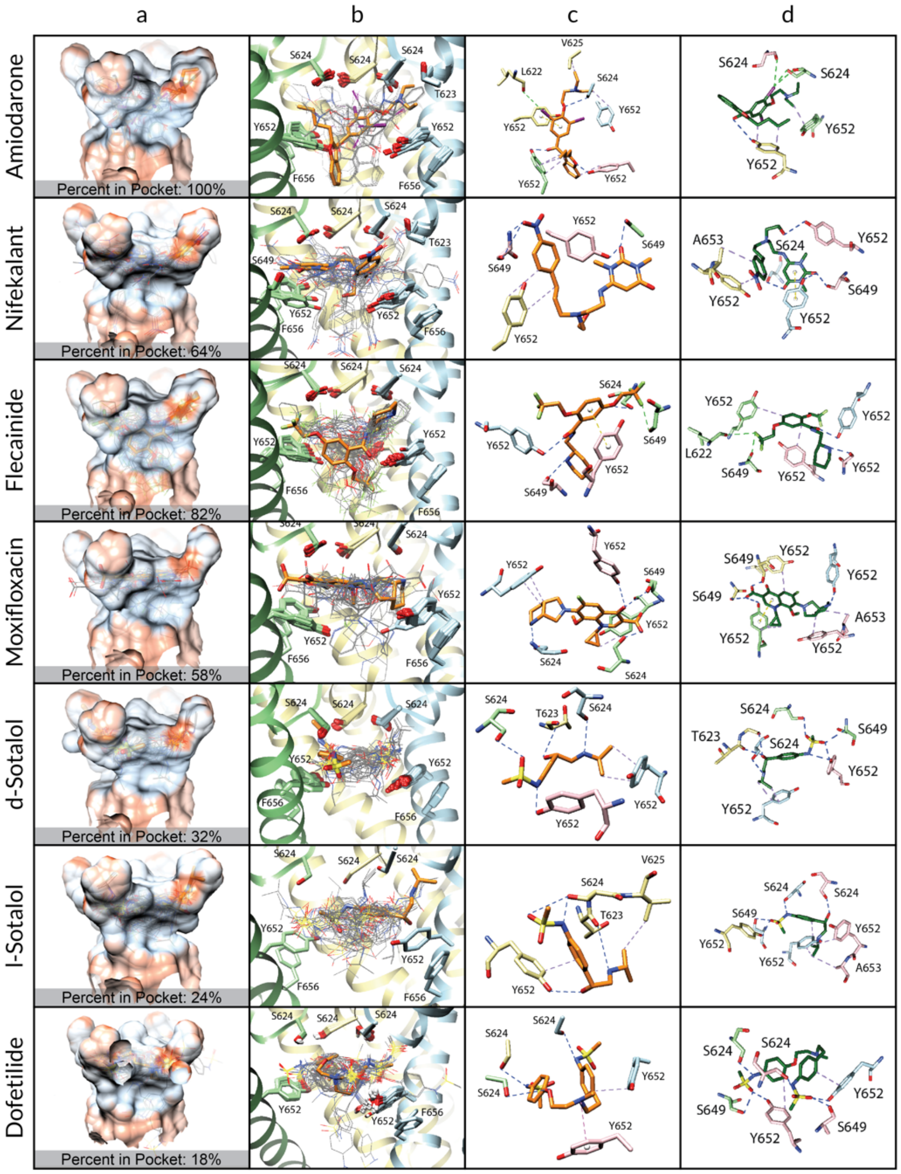

Supplement Figure 2. Y652A Open-State hERG Channel Docking with Cationic or Zwitterionic Drugs. Rosetta docking results of Y652A hERG channel mutant in open state with cationic or zwitterionic forms of each drug. Chain A is colored pink, chain B is blue, chain C is yellow, chain D is green, and ligand C atoms are gray. Top pose from largest and second largest clusters are in stick form and are orange and dark green, respectively. O atoms – red, N – blue, S – yellow, I – violet. H atoms not shown for clarity. Panel A shows top 50 poses in gray sorted by lowest interface score. Panel B highlights representative poses identified as the lowest energy pose from the largest and second largest cluster, respectively. PLIP-identified interactions are indicated by dashed lines. Halogen bonds are colored green, hydrogen bonds in blue, cation-pi in pink, pi-stacking in yellow, hydrophobic interactions in pale purple.

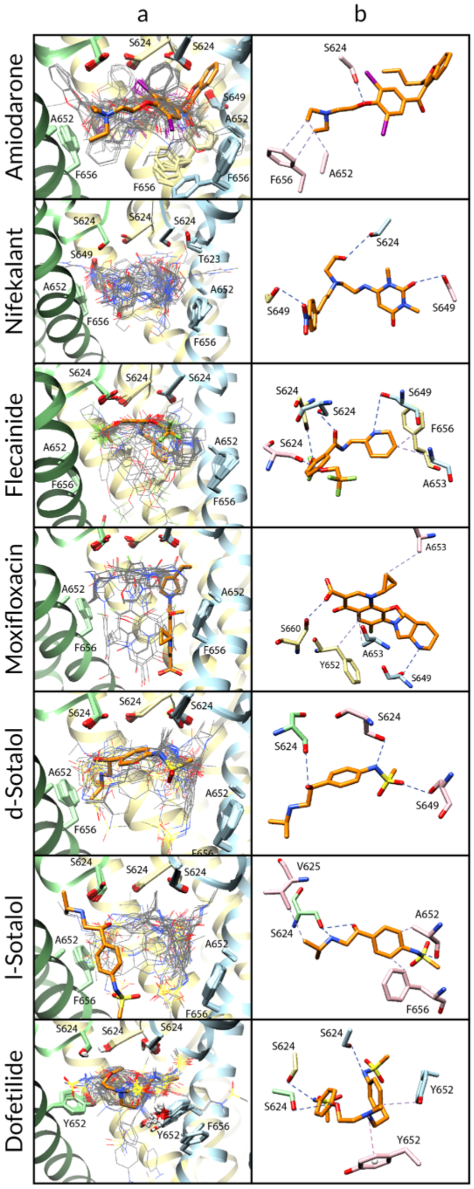

Supplement Figure 3. F656A Open-State hERG Channel Docking with Cationic or Zwitterionic Drugs. Rosetta docking results of F656A hERG channel mutant in open state with cationic or zwitterionic forms of each drug. Chain A is colored pink, chain B is blue, chain C is yellow, chain D is green, and ligand C atoms are gray. Top pose from largest and second largest clusters are in stick form and are orange and dark green, respectively. O atoms – red, N – blue, S – yellow, I – violet. H atoms not shown for clarity. Panel A shows top 50 poses in gray sorted by lowest interface score. Panel B highlights representative poses identified as the lowest energy pose from the largest and second largest cluster, respectively. PLIP-identified interactions are indicated by dashed lines. Halogen bonds are colored green, hydrogen bonds in blue, cation-pi in pink, pi-stacking in yellow, hydrophobic interactions in pale purple.

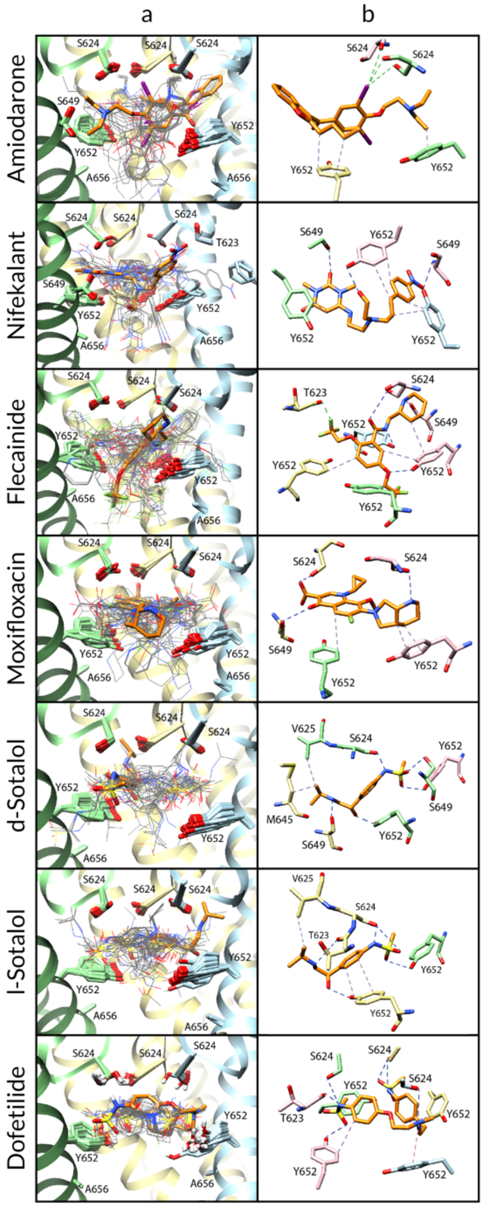

Supplement Figure 4. Y652A/F656A Open-State hERG Channel Docking with Cationic or Zwitterionic Drugs. Rosetta docking results of Y652A/F656A hERG channel mutant in open state with cationic or zwitterionic forms of each drug. Chain A is colored pink, chain B is blue, chain C is yellow, chain D is green, and ligand C atoms are gray. Top pose from largest and second largest clusters are in stick form and are orange and dark green, respectively. O atoms – red, N – blue, S – yellow, I – violet. H atoms not shown for clarity. Panel A shows top 50 poses in gray sorted by lowest interface score. Panel B highlights representative poses identified as the lowest energy pose from the largest and second largest cluster, respectively. PLIP-identified interactions are indicated by dashed lines. Halogen bonds are colored green, hydrogen bonds in blue, cation-pi in pink, pi-stacking in yellow, hydrophobic interactions in pale purple.

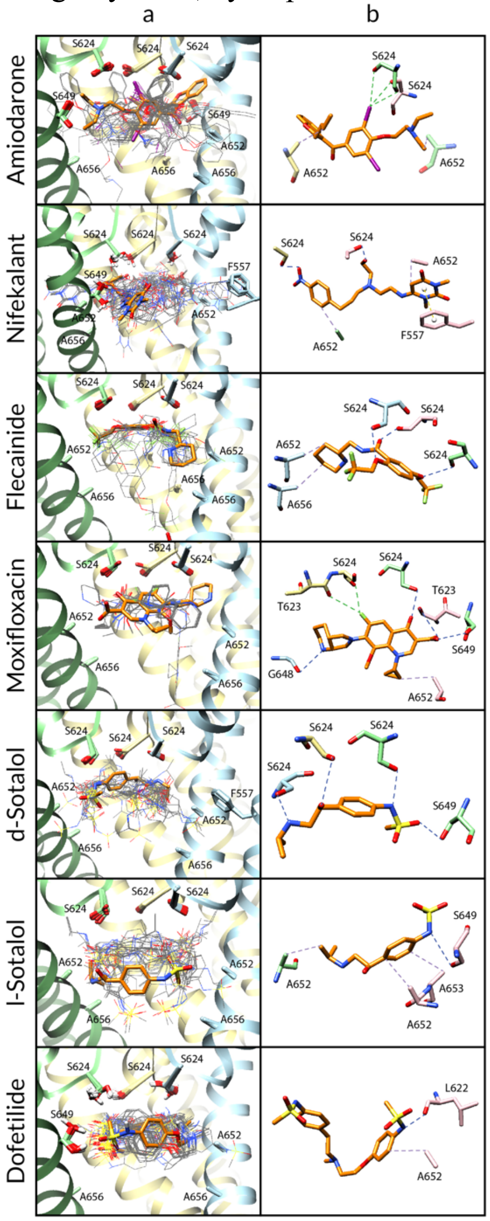

Supplement Figure 5. Closed-State hERG Channel Docking with Cationic or Zwitterionic Drugs. Rosetta docking results of closed-state WT (5) hERG channel interactions with cationic or zwitterionic drugs. Chain A is colored pink, chain B is blue, chain C is yellow, chain D is green, and ligand C atoms are gray or orange (for top pose). O atoms – red, N – blue, S – yellow, I – violet. H atoms not shown for clarity. Panel A shows top 50 poses colored by position. Within pore is dark magenta, fenestration is orange, intracellular gate is cyan, and membrane is pale pink. Panel B shows top 50 poses in gray sorted by lowest interface score. Panel C highlights representative poses identified as the lowest energy pose from the largest cluster. PLIP-identified interactions are indicated by dashed lines. Halogen bonds are colored green, hydrogen bonds in blue, cation-pi in pink, pi-stacking in yellow, hydrophobic interactions in pale purple.

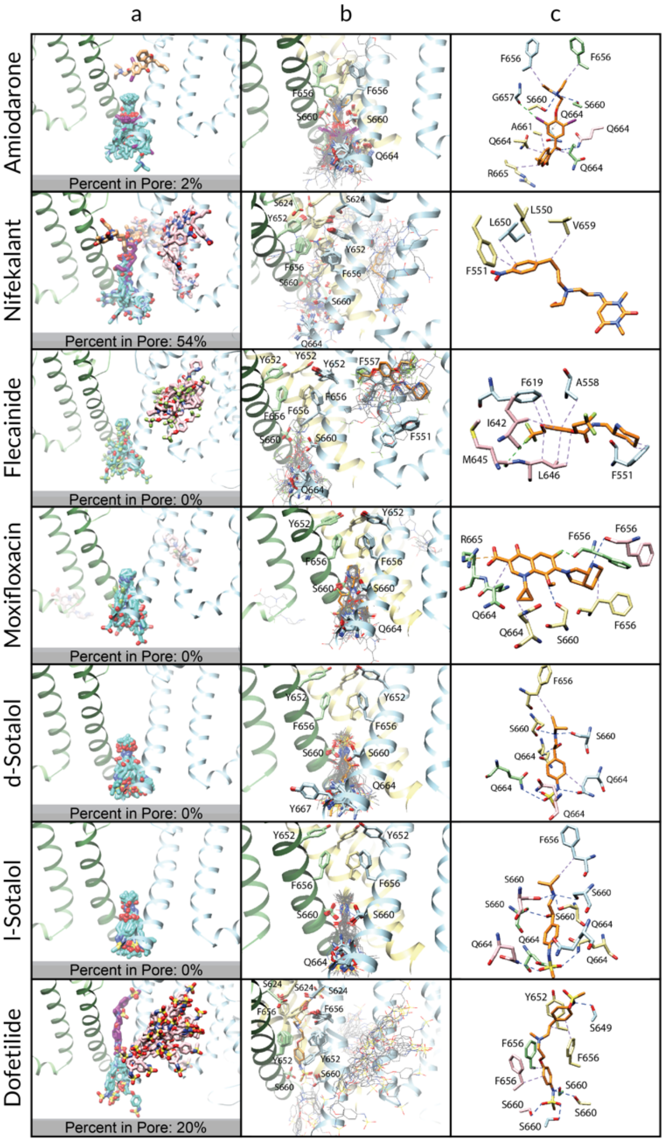

Supplement Figure 6. Amiodarone – Probability Density and B&W Plot. Left panels are box-and-whisker plots of top 50 interface scores (measured in Rosetta Energy Units) of amiodarone with each protein model. To compare each state to the open-state, wild-type, two sample for variance F-tests were done, followed by the corresponding t-tests assuming equal or unequal variances. Significance at p=0.5 is indicated by * and at p=0.001 by *** are indicated above their respective data sets. Right panels are probability density charts plotting probability versus interface score. Open-state wild-type is red, open-state F656A mutant is orange, open-state Y652A is yellow, open-state F656A/Y652A is green, and closed-state is blue.

Neutral Amiodarone

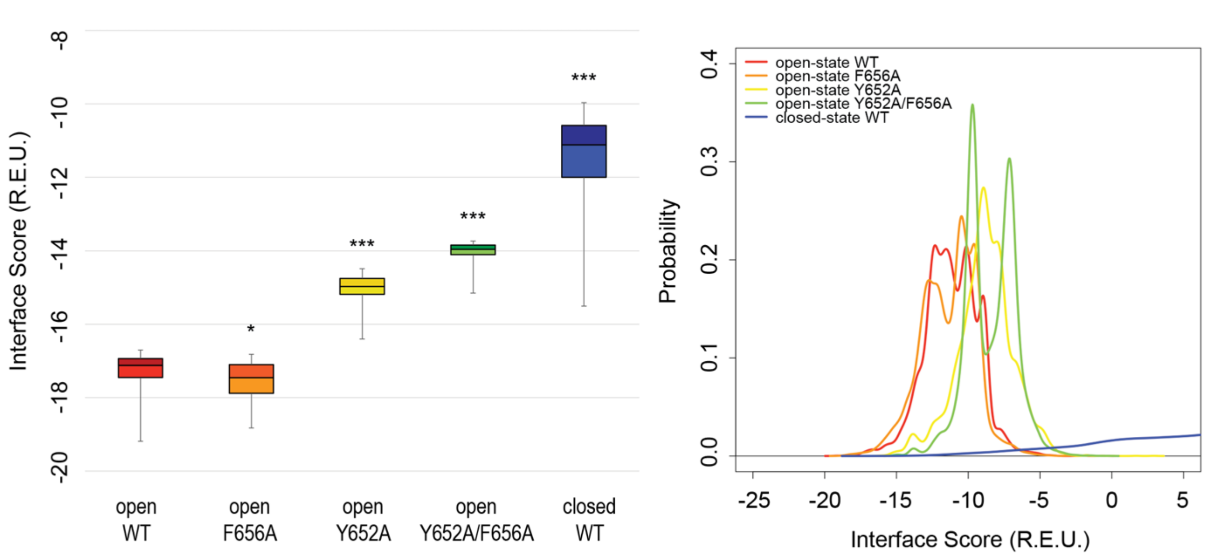

Cationic Amiodarone

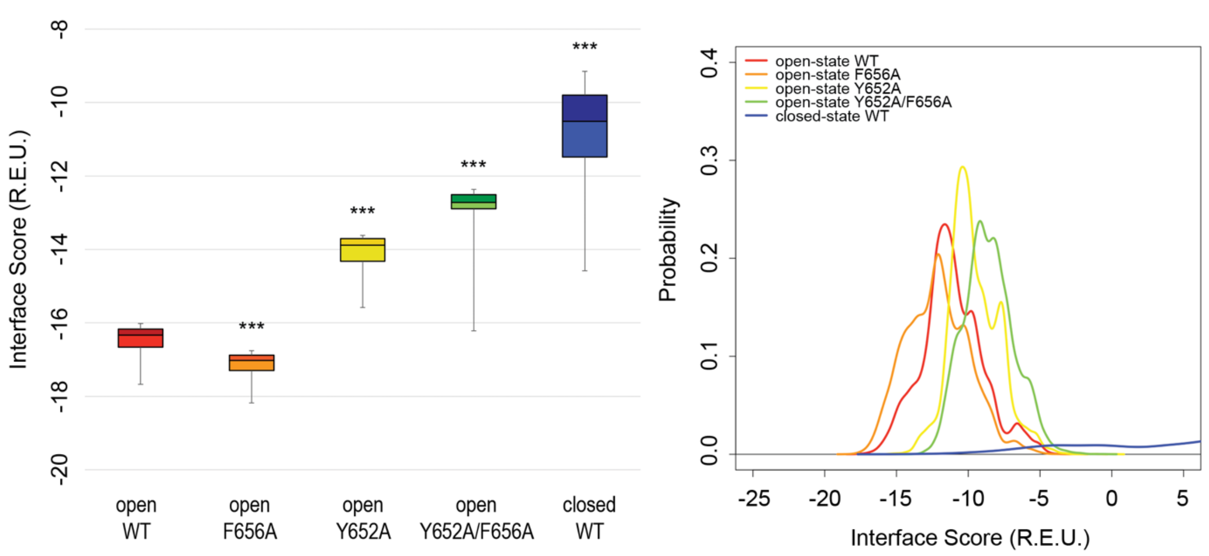

Supplement Figure 7. Nifekalant – Probability Density and B&W Plot. Left panels are box-and-whisker plots of the top 50 interface scores (measured in Rosetta Energy Units) of nifekalant with each protein model. To compare each state to the open WT, two sample for variance F-tests were done, followed by the corresponding t-tests assuming equal or unequal variances. Significance at p=0.01 is indicated by ** and at p=0.001 by *** are indicated above their respective data sets. Right panels are probability density charts plotting probability versus interface score of top 10,000. Open-state wild-type is red, open-state F656A mutant is orange, open-state Y652A is yellow, open-state F656A/Y652A is green, and closed-state is blue.

Neutral Nifekalant

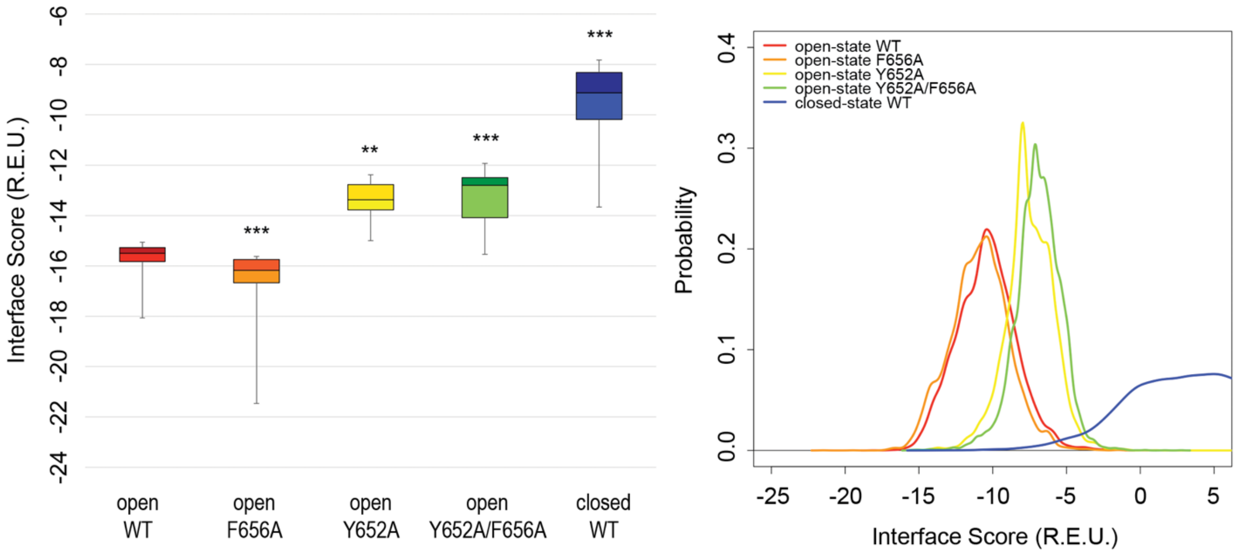

Cationic Nifekalant

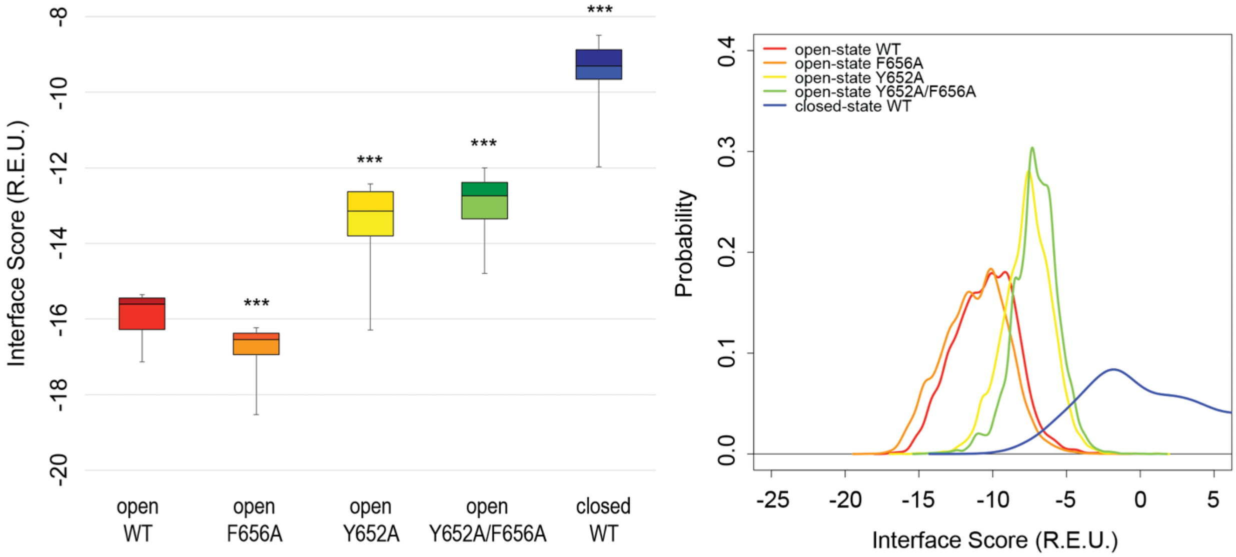

Supplement Figure 8. Flecainide – Probability Density and B&W Plot. Left panels are box-and-whisker plots of the top 50 interface scores (measured in Rosetta Energy Units) of flecainide with each protein model. To compare each state to the open WT, two sample for variance F-tests were done, followed by the corresponding t-tests assuming equal or unequal variances. Significance at p=0.001 is indicated by *** are indicated above their respective data sets. Right panels are probability density charts plotting probability versus interface score of top 10,000. Open-state wild-type is red, open-state F656A mutant is orange, open-state Y652A is yellow, open-state F656A/Y652A is green, and closed-state is blue.

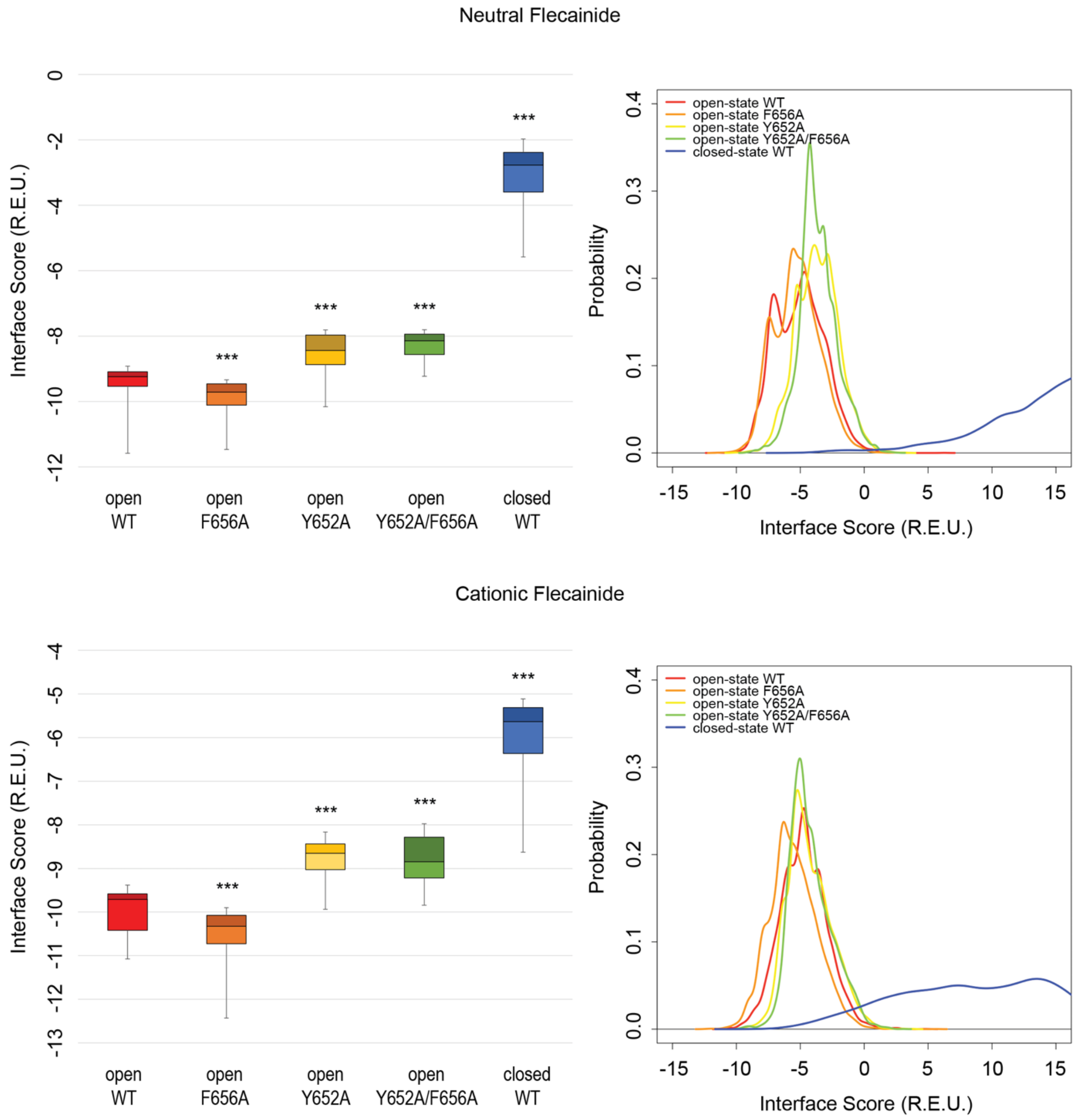

Supplement Figure 9. Moxifloxacin – Probability Density and B&W Plot. Left panels are box-and-whisker plots of the top 50 interface scores (measured in Rosetta Energy Units) of moxifloxacin with each protein model. To compare each state to the open WT, two sample for variance F-tests were done, followed by the corresponding t-tests assuming equal or unequal variances. Significance at p=0.05 is indicated by *, at p=0.01 by **, and at p=0.001 by *** are indicated above their respective data sets. Right panels are probability density charts plotting probability versus interface score of top 10,000. Open-state wild-type is red, open-state F656A mutant is orange, open-state Y652A is yellow, open-state F656A/Y652A is green, and closed-state is blue.

Neutral Moxifloxacin

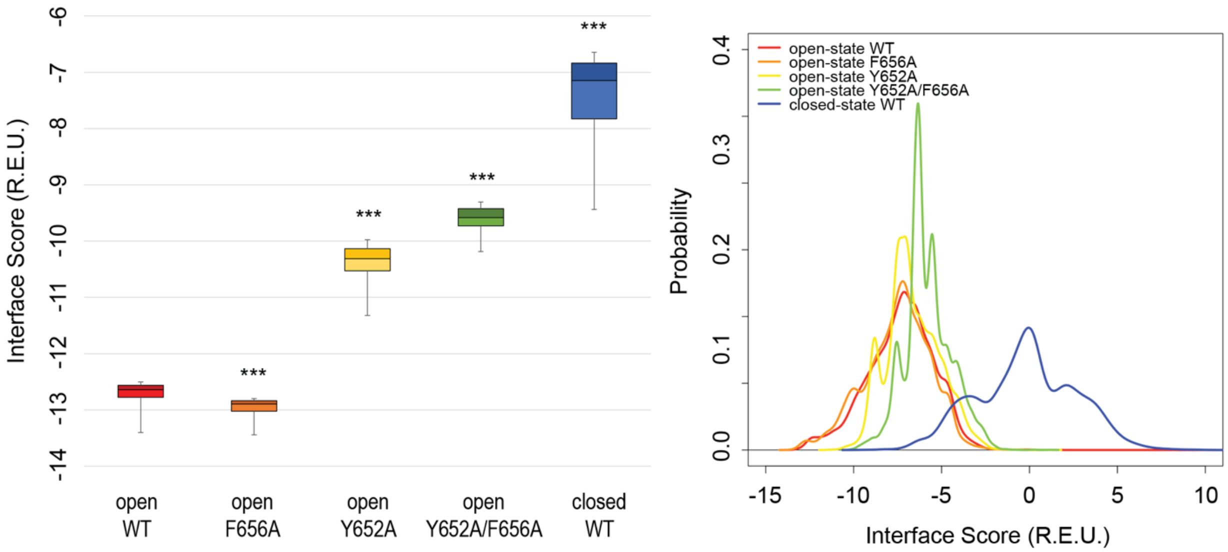

Zwitterionic Moxifloxacin

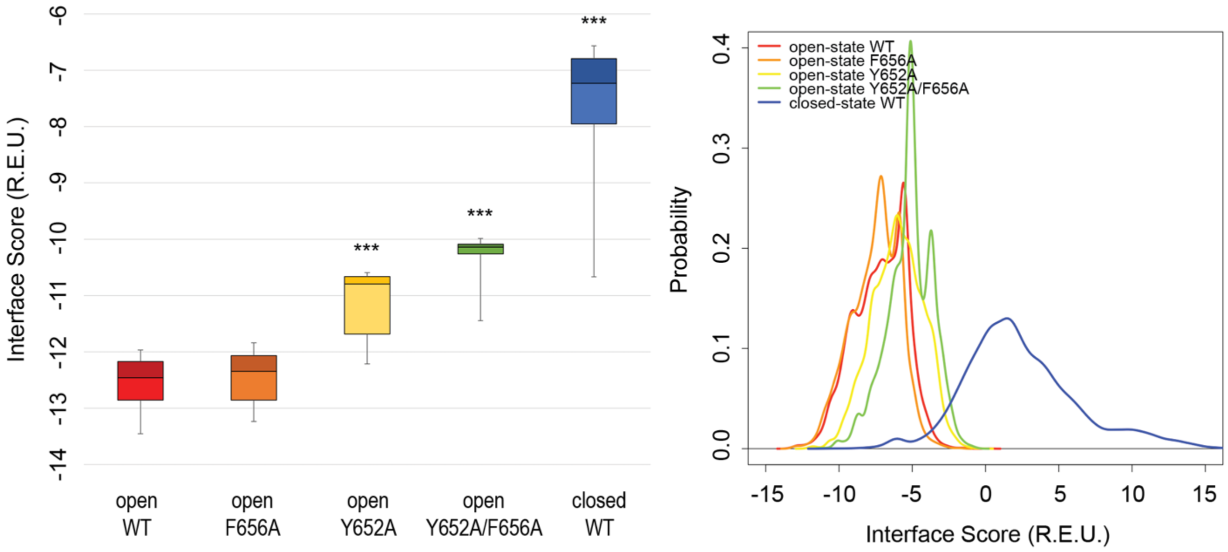

Supplement Figure 10. d-Sotalol – Probability Density and B&W Plot. Left panels are box-and-whisker plots of the top 50 interface scores (measured in Rosetta Energy Units) of d-sotalol with each protein model. To compare each state to the open WT, two sample for variance F-tests were done, followed by the corresponding t-tests assuming equal or unequal variances. Significance at p=0.05 is indicated by *, at p=0.01 by **, and at p=0.001 by *** are indicated above their respective data sets. Right panels are probability density charts plotting probability versus interface score of top 10,000. Open-state wild-type is red, open-state F656A mutant is orange, open-state Y652A is yellow, open-state F656A/Y652A is green, and closed-state is blue.

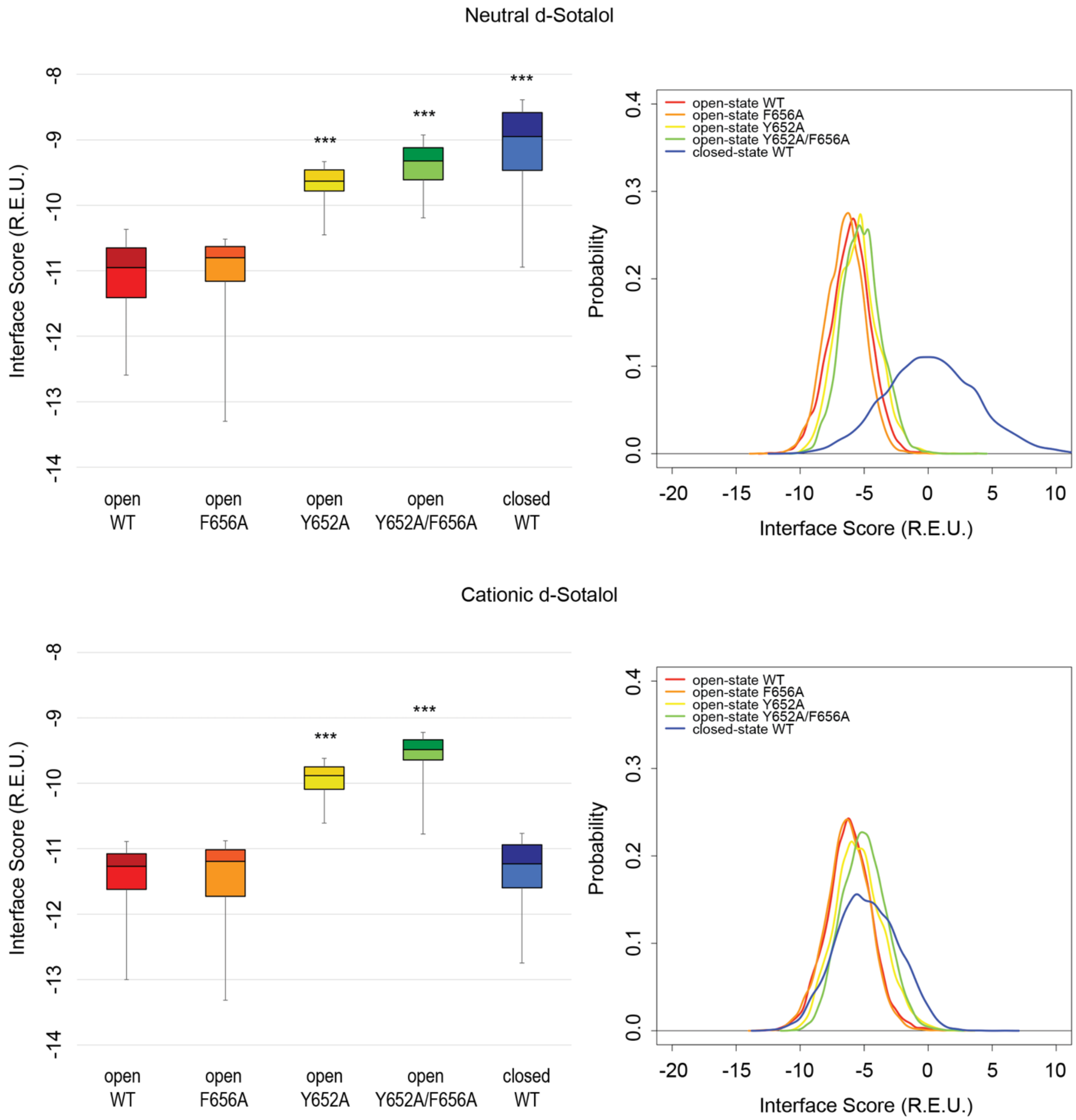

Supplement Figure 11. l-Sotalol – Probability Density and B&W Plot. Left panels are box-and-whisker plots of the top 50 interface scores (measured in Rosetta Energy Units) of l-sotalol with each protein model. To compare each state to the open WT, two sample for variance F-tests were done, followed by the corresponding t-tests assuming equal or unequal variances. Significance at p=0.05 is indicated by *, at p=0.01 by **, and at p=0.001 by *** are indicated above their respective data sets. Right panels are probability density charts plotting probability versus interface score of top 10,000. Open-state wild-type is red, open-state F656A mutant is orange, open-state Y652A is yellow, open-state F656A/Y652A is green, and closed-state is blue.

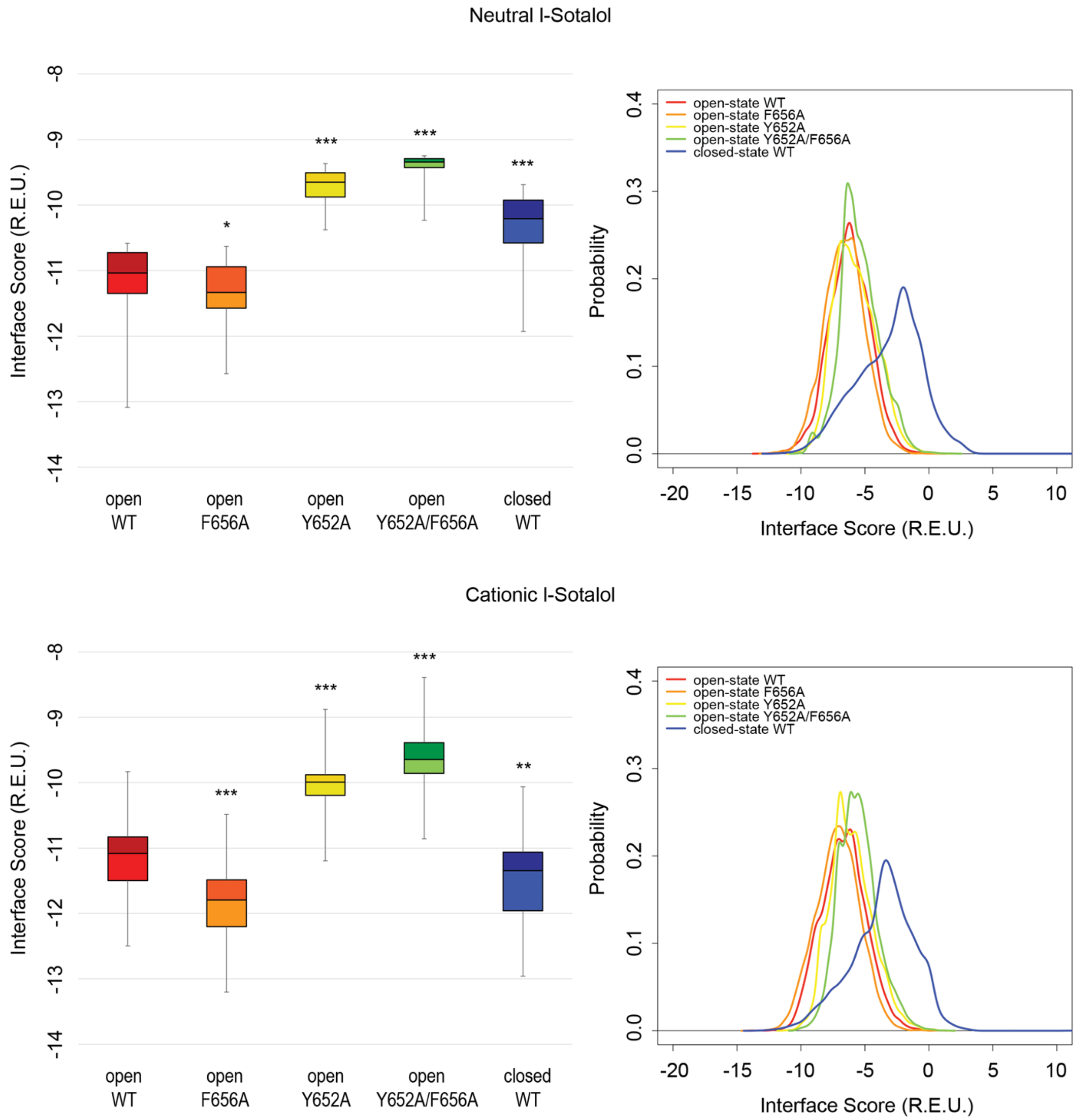

Supplement Figure 12. Dofetilide – Probability Density and B&W Plot. Left panels are box-and-whisker plots of the top 50 interface scores (measured in Rosetta Energy Units) of dofetilide with each protein model. To compare each state to the open WT, two sample for variance F-tests were done, followed by the corresponding t-tests assuming equal or unequal variances. Significance at p=0.05 is indicated by *, at p=0.01 by **, and at p=0.001 by *** are indicated above their respective data sets. Right panels are probability density charts plotting probability versus interface score of top 10,000. Open-state wild-type is red, open-state F656A mutant is orange, open-state Y652A is yellow, open-state F656A/Y652A is green, and closed-state is blue.

Neutral Dofetilide

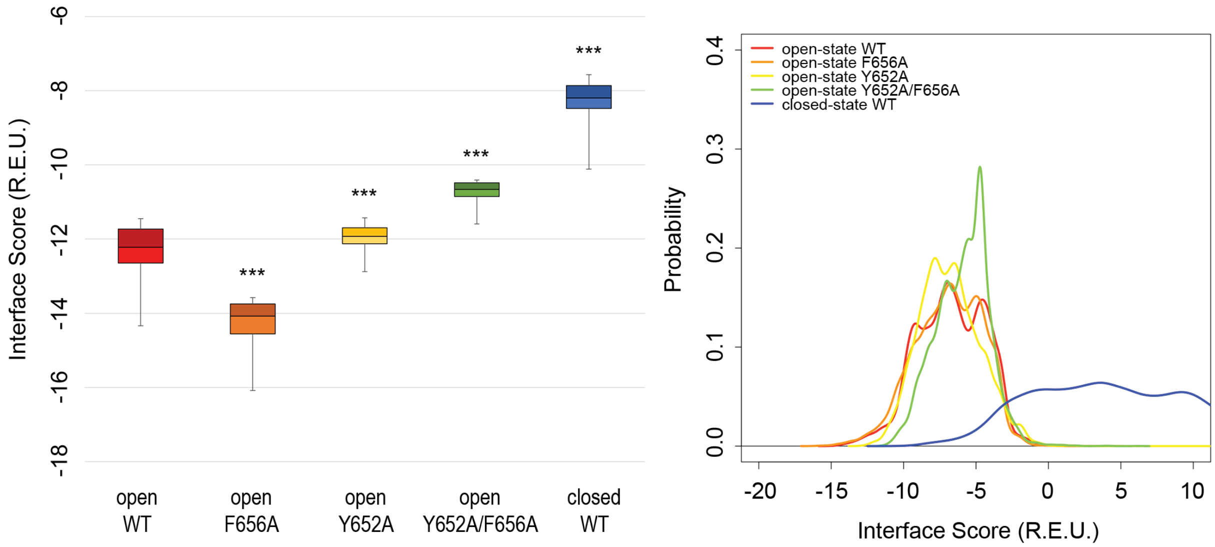

Cationic Dofetilide

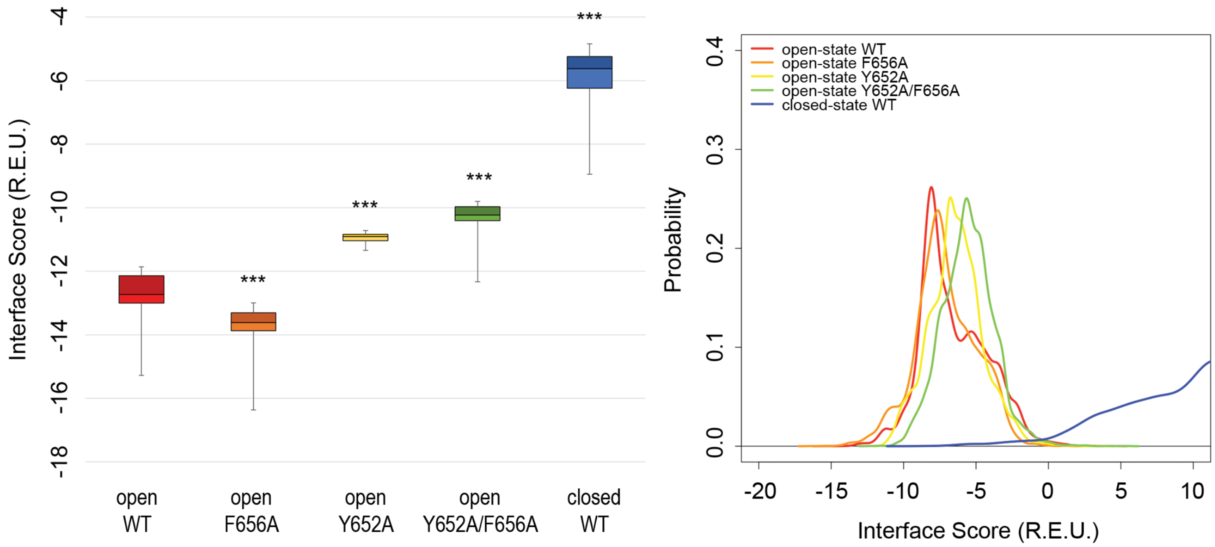

Supplement Figure 13. Fenestration Regions. Surface representations of open (blue) and closed (red) state WT hERG structures with fenestration regions (FR) available as a result of drug (white) docking interactions.

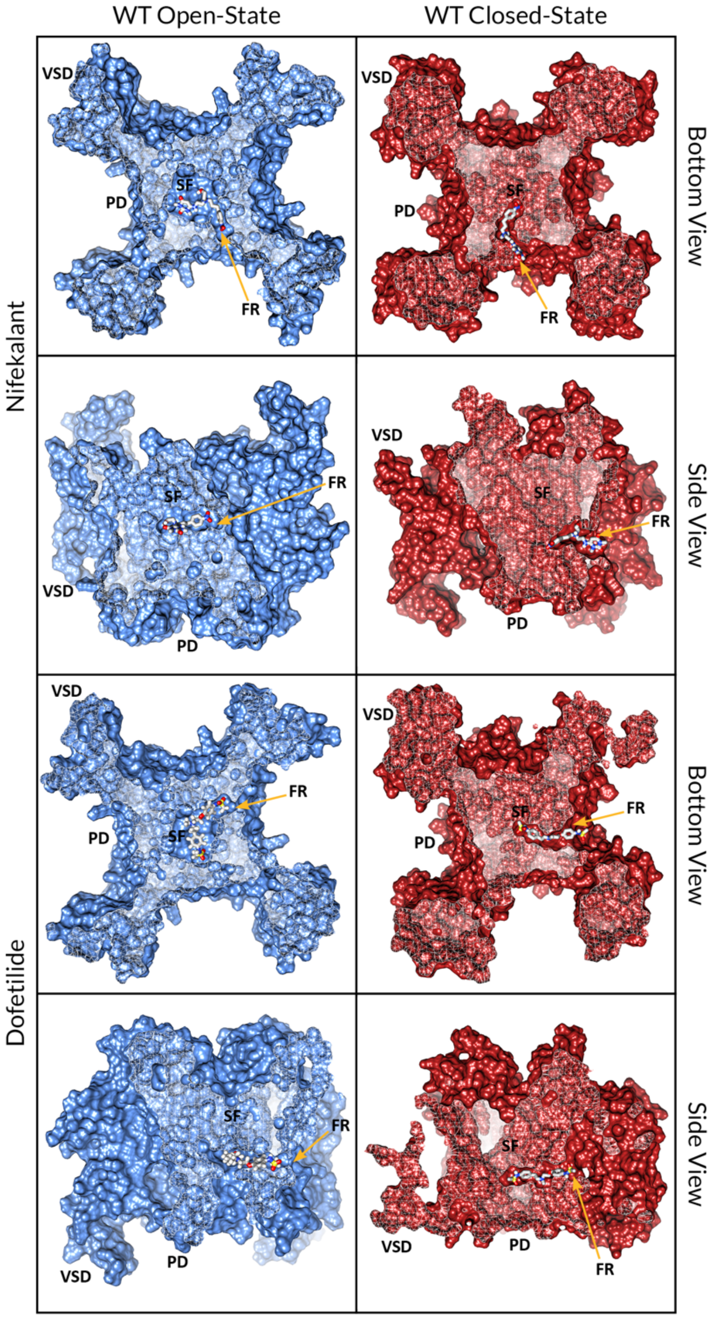

Supplement Figure 14. Interface Score vs RMSD Plots. Interface score plotted against root-mean-square deviation from the reference pose of the top 1,000 poses by interface score of the top 10,000 poses by total score. Clustering was done only on the top 50 poses by interface score as indicated by horizontal line. Black dots below line are unclustered poses within the top 50.

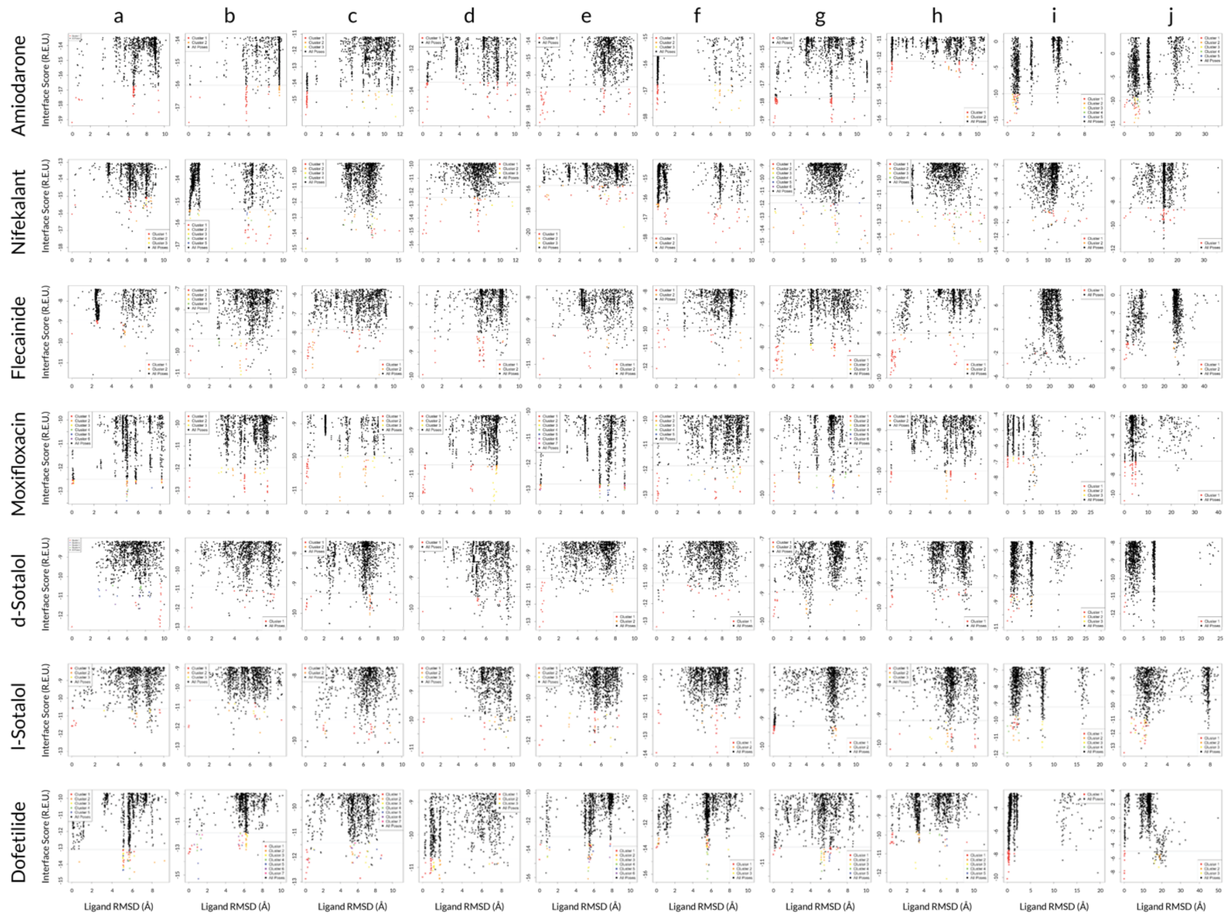

Supplement Script 1. XML cryo-EM refinement protocol

```
<ROSETTASCRIPTS>
  <SCOREFXNS>
    <ScoreFunction name="dens" weights="beta_cart">
      <Reweight scoretype="elec_dens_fast" weight="35.0"/>
      <Set
scale_sc_dens_byres="R:0.76,K:0.76,E:0.76,D:0.76,M:0.76,C:0.81,Q:0.81,H:0.81,N:0.81,T:0.81
,S:0.81,Y:0.88,W:0.88,A:0.88,F:0.88,P:0.88,I:0.88,L:0.88,V:0.88"/>
    </ScoreFunction>
  </SCOREFXNS>

  <MOVERS>
     <SetupForDensityScoring name="setupdens"/>
     <LoadDensityMap name="loaddens"
mapfile="/share/yarovlab/amemigh/projects/input/nav1.4-fitting/emd_9617.map"/>
     <FastRelax name="relaxcart" scorefxn="dens" repeats="2" cartesian="1"/>
  </MOVERS>

  <PROTOCOLS>
    <Add mover="setupdens"/>
    <Add mover="loaddens"/>
    <Add mover="relaxcart"/>
  </PROTOCOLS>
  <OUTPUT scorefxn="dens"/>

</ROSETTASCRIPTS>
```

Supplement Script 2. XML homology modeling protocol

```
<ROSETTASCRIPTS>
<SCOREFXNS>
   <ScoreFunction name="stage1" weights="score3" symmetric="1">
      <Reweight scoretype="atom_pair_constraint" weight="0.5"/>
   </ScoreFunction>
   <ScoreFunction name="stage2" weights="score4_smooth_cart" symmetric="1">
      <Reweight scoretype="atom_pair_constraint" weight="0.5"/>
   </ScoreFunction>
   <ScoreFunction name="fullatom" weights="ref2015_cart" symmetric="1">
      <Reweight scoretype="atom_pair_constraint" weight="0.5"/>
   </ScoreFunction>
   <ScoreFunction name="ref2015" weights="ref2015" symmetric="1">
   </ScoreFunction>
</SCOREFXNS>
<MOVERS>
   <Hybridize name="hybridize" stage1_scorefxn="stage1" stage2_scorefxn="stage2"
fa_scorefxn="fullatom" batch="1" stage1_increase_cycles="1.0" stage2_increase_cycles="1.0">
      <Template pdb="{path}/herg-template-1D.pdb" cst_file="AUTO" weight="1.000"
symmdef="{path}/herg-template-1D.symm"/>
      Fragments three_mers="aaherg-1D_03_05.200_v1_3" nine_mers="aaherg-
1D_09_05.200_v1_3" />
   </Hybridize>
   <FastRelax name="relax" scorefxn="ref2015"  />
</MOVERS>
<PROTOCOLS>
   <Add mover="hybridize"/>
</PROTOCOLS>
<OUTPUT scorefxn="ref2015" />
</ROSETTASCRIPTS>
```

Supplement Script 3. XML docking protocol

```
<ROSETTASCRIPTS>
   <SCOREFXNS>
      <ligand_soft_rep weights="ligand_soft_rep">
         <Reweight scoretype="fa_elec" weight="0.42"/>
         <Reweight scoretype="hbond_bb_sc" weight="1.3"/>
         <Reweight scoretype="hbond_sc" weight="1.3"/>
         <Reweight scoretype="rama" weight="0.2"/>
      </ligand_soft_rep>

      <hard_rep weights=ligand>
         <Reweight scoretype="fa_intra_rep" weight="0.004"/>
         <Reweight scoretype="fa_elec" weight="0.42"/>
         <Reweight scoretype="hbond_bb_sc" weight="1.3"/>
         <Reweight scoretype="hbond_sc" weight="1.3"/>
         <Reweight scoretype="rama" weight="0.2"/>
      </hard_rep>
   </SCOREFXNS>
   <LIGAND_AREAS>
      <docking_sidechain chain="X" cutoff="7.0" add_nbr_radius="true" all_atom_mode="true"
minimize_ligand="10"/>
      <final_sidechain chain="X" cutoff="7.0" add_nbr_radius="true" all_atom_mode="true"/>
      <final_backbone chain="X" cutoff="7.0" add_nbr_radius="false" all_atom_mode="true"
Calpha_restraints="0.3"/>
   </LIGAND_AREAS>

   <INTERFACE_BUILDERS>
      <side_chain_for_docking ligand_areas="docking_sidechain"/>
      <side_chain_for_final ligand_areas="final_sidechain"/>
      <backbone ligand_areas="final_backbone" extension_window="3"/>
   </INTERFACE_BUILDERS>

   <MOVEMAP_BUILDERS>
      <docking sc_interface="side_chain_for_docking" minimize_water="true"/>
      <final sc_interface="side_chain_for_final" bb_interface="backbone"
minimize_water="true"/>
   </MOVEMAP_BUILDERS>

   <SCORINGGRIDS ligand_chain="X" width="15">
      <vdw grid_type="ClassicGrid" weight="1.0"/>
   </SCORINGGRIDS>

   <MOVERS>
      <Transform name="transform" chain="X" box_size="5.0" move_distance="0.1" angle="5"
cycles="1000" repeats="1" temperature="5" initial_perturb="5.0"/>
```

```
        <HighResDocker name="high_res_docker" cycles="6" repack_every_Nth="3"
scorefxn="ligand_soft_rep" movemap_builder="docking"/>
        <FinalMinimizer name="final" scorefxn="hard_rep" movemap_builder="final"/>
        <InterfaceScoreCalculator name="add_scores" chains="X" scorefxn="hard_rep"
compute_grid_scores="0"/>
        AddJobPairData name="system_name" key="system_name" value_type="string"
value_from_ligand_chain="X"

        <ParsedProtocol name="low_res_dock">
          <Add mover_name="transform"/>
        </ParsedProtocol>

        <ParsedProtocol name="high_res_dock">
          <Add mover_name="high_res_docker"/>
          <Add mover_name="final"/>
        </ParsedProtocol>

        <ParsedProtocol name="reporting">
          <Add mover_name="add_scores"/>
          <Add mover_name="system_name"/>
        </ParsedProtocol>
    </MOVERS>

    <PROTOCOLS>
        <Add mover_name="low_res_dock"/>
        <Add mover_name="high_res_dock"/>
        <Add mover_name="reporting"/>
    </PROTOCOLS>

</ROSETTASCRIPTS>
```

Supplement Script 4. Tcl clustering parameters extraction

```
proc getz {l d} {
   set com1 [measure center $l weight mass]
   set com2 [measure center $d weight mass]
   set dz [vecsub $com2 $com1]
   return [lindex $dz 2]
}

proc theta {s c} {
      #Get tilt angle between drug and z
      #special arctan
   return [expr atan2($s,$c)]
}

proc phi {v} {
      #Get azimuth angle between drug and x
   set x [lindex $v 0]
   set y [lindex $v 1]
   return [expr atan2($y,$x)]
}

proc rad2deg {a} {
      set PI 3.14159
   return [expr $a * (180.0/$PI)]
}

set moln 0

mol new top50_1.pdb type pdb waitfor all

for { set j 2 } { $j <= 50 } { incr j 1 } {
   animate read pdb top50_${j}.pdb beg 0 end -1 skip 1  waitfor all $moln
   }

set outfile [open ligand-tumble_closed_nif1.dat w]
set nf [molinfo top get numframes]

      puts "number of frames $nf"

set sf [atomselect top "sequence SVGFG"]
set drug [atomselect top "chain X"]
set PI 3.14159
   return [expr $a * (180.0/$PI)]
}
```

```
set moln 0

mol new top50_1.pdb type pdb waitfor all

for { set j 2 } { $j <= 50 } { incr j 1 } {
   animate read pdb top50_${j}.pdb beg 0 end -1 skip 1  waitfor all $moln
   }

set outfile [open ligand-tumble_closed_nif1.dat w]
set nf [molinfo top get numframes]

      puts "number of frames $nf"

set sf [atomselect top "sequence SVGFG"]
set drug [atomselect top "chain X"]

set H [atomselect top "chain X and name O2"]
set T [atomselect top "chain X and name O3"]

for { set i 0 } { $i <= $nf } { incr i } {

            $sf frame $i
            $drug frame $i
            set zpos [getz $sf $drug]

            $H frame $i
            set Hcoor [lindex [$H get {x y z}] 0]
            $T frame $i
            set Tcoor [lindex [$T get {x y z}] 0]

            set vlen [ veclength [vecsub $Hcoor $Tcoor]]

            set vdrug [vecnorm [vecsub $Hcoor $Tcoor]]

            set vz {0 0 1}
            set rotvec [veccross $vdrug $vz]
            set sine [veclength $rotvec]
            set cosine [vecdot $vdrug $vz]
            set th [theta $sine $cosine]
            set ph [phi $vdrug]
            if {$ph < 0 } {
               set ph [expr $ph +360]
            }

            puts $outfile [format "%6d\t%8f\t%8f\t%8f\t%8f\t%8f\t%8f" $i $zpos $sine $cosine
$th $ph $vlen]
```

```
    }

  close $outfile

  $sf delete
  $drug delete
  $H delete
  $T delete

  mol delete all
```

Supplement Script 5. R clustering algorithm

```
#### RScript to Calculate Symmetric RMSD
library(tidyverse)
library(animation)
library(rlist)
### Read in data file and cut
setwd("/Users/aiyana/Box/Work-In-Progress/workInProgress/l-sotalol/dock-herg-closed-
lsot1/top-50")
dft <- read.table("ligand-tumble_closed_sotl1.dat",header=F)
temp <- dft[,c(2,5,7)]
data <- head(temp,-1)
data_scaled <- scale(data)

### Find max, min and diffs of each column
colMax <- function(data) sapply(data,max)
maxs <- colMax(data)
colMin <- function(data) sapply(data,min)
mins <- colMin(data)
diffs = maxs - mins

### Initialize variables
n_row = nrow(data)
n_col = ncol(data)
vars <- array(0,c(nrow(data),ncol(data),nrow(data)))
sims <- array(0,c(nrow(data),nrow(data)))
counts <- array(0,c(ncol(sims)))
cutoff = 0.05
size_min = 3

### Calculate squares of deviation for each variable
ref = 1
while (ref <= n_row){
  for (row in 1:n_row) {
    for (col in 1:n_col) {
      vars[row,col,ref] = ((data[ref,col]-data[row,col])/diffs[col])^2
```

```
    }
  }
  ref = ref + 1
}

### Calculate similarity (squareroot of sums)
ref = 1
sums = 0
while (ref <= n_row) {
  for (row in 1:n_row) {
    for (col in 1:n_col) {
      sums = sums + vars[row,col,ref]
    }
    sims[row,ref] = sqrt(sums)
    if (sims[row,ref] <= cutoff) {

    }
    sums = 0
  }
  ref = ref + 1
}

### Count number of neighbors within cutoff
neighbors <- list()
for (col in 1:ncol(sims)) {
  counts[col] = sum(sims[,col] <= cutoff)
  nbrs <- c()
  count = 0
  for (row in 1:nrow(sims)) {
    if (sims[row,col] <= cutoff) {
      count = count + 1
      nbrs[count] <- row
    }
  }
  neighbors[[col]] <- nbrs
}

### Find unique first elements
matches <- c()
count2 = 0
for (row in 1:(length(neighbors)-1)) {
  temp1 <- vector(,length(neighbors[[row]]))
  temp1 <- neighbors[[row]]
  for (second in (row+1):length(neighbors)) {
    temp2 <- vector(,length(neighbors[[second]]))
    temp2 <- neighbors[[second]]
```

```
    if (temp1[1] == temp2[1]) {
      count2 = count2 + 1
      matches[count2] <- second
    }
  }
}
matches_cull <- matches[!duplicated(matches)]
matches_sort <- sort(matches_cull, decreasing = TRUE)

for(values in 1:length(matches_sort)) {
  neighbors[[matches_sort[values]]] <- NULL
}

### Set min size of clusters
for(rows in length(neighbors):1) {
  if(length(neighbors[[rows]])<=size_min){
    neighbors[[rows]] <- NULL
  }
}

### Set lowest unique cluster as centroids
clusters <- array(0,as.integer(length(neighbors)))
for(i in 1:length(neighbors)) {
  temp1 <- vector(,length(neighbors[[i]]))
  temp1 <- neighbors[[i]]
  clusters[i] <- temp1[1]
}

### Build centroid array with scaled data
centroid <- array(0,c(length(clusters),ncol(data_scaled)))
for (col in 1:ncol(data_scaled)) {
  for (row in 1:length(clusters)) {
    centroid[row,col] <- data_scaled[clusters[row],col]
  }
}

### Kmeans optimization of clusters
op <- kmeans(data_scaled,centroid)
print(op)

### Find largest cluster number
op_cluster <- array(op$cluster)
op_size <- array(op$size)
largest_cluster_num <- which.max(op_size)

### Find lowest energy structure from largest cluster
```

```
lowest_energy <- 0
for(pose in 1:length(op_cluster)) {
  if(op_cluster[pose]==largest_cluster_num) {
    lowest_energy = pose
    break
  }
}

### Populate array with poses of largest cluster
largest_cluster <- vector(mode = "integer",length = max(op_size))
i=1
#pose=1
##print(length(op_cluster))
for(pose in 1:length(op_cluster)) {
  if(op_cluster[pose]==largest_cluster_num) {
    #cat("position, cluster, index: ", pose, op_cluster[pose], i)
    largest_cluster[i] = pose
    i=i+1
  }
}

### Create list with outliers removed
noOutliers <- NULL
for (row in 1:length(neighbors)) {
  noOutliers <- append(noOutliers, neighbors[[row]])
  }
noOutliers <- unique(noOutliers)

#print(op_cluster)

### Print Results for largest cluster only
### cat("The largest cluster has", op_size[5], "members.")
### cat("The lowest energy (representative) structure from the largest cluster is",
paste0(lowest_energy,"."))
### cat("The members of this cluster are", largest_cluster)

### Create List of all clusters, print results
'%!in%' <- function(x,y)!('%in%'(x,y))
all_clusters <- vector(mode = "list", length = length(op_size))
for(index in 1:length(op_size)){
    all_clusters[[index]] <- which(op_cluster %in% index)
    for(element in 1:length(all_clusters[[index]])) {
      if(all_clusters[[index]][element] %!in% noOutliers){
        all_clusters[[index]][element] <- 0
      }
    }
```

```
}
all_clusters <- lapply(all_clusters,function(x) x[x!=0]) #How to remove zero elements?
sorted_clusters <- all_clusters[order(sapply(all_clusters,length),decreasing=T)]
print(sorted_clusters)
paste(sorted_clusters, collapse = ", ")
#testing <- paste(sorted_clusters[1], sep = ",")
#gsub()
```
